# Diabetes exacerbates SARS-CoV-2 replication through ineffective pulmonary interferon responses, delayed cell-mediated immunity, and disruption of leptin signaling

**DOI:** 10.3389/fcimb.2025.1513687

**Published:** 2025-03-07

**Authors:** Côme J. Thieulent, Udeni B. R. Balasuriya, Anna Tseng, Nicholas A. Crossland, Jacqueline M. Stephens, Wellesley Dittmar, Jaroslaw Staszkiewicz, Juergen A. Richt, Mariano Carossino

**Affiliations:** ^1^ Department of Pathobiological Sciences, School of Veterinary Medicine, Louisiana State University, Baton Rouge, LA, United States; ^2^ Louisiana Animal Disease Diagnostic Laboratory (LSU Diagnostics), School of Veterinary Medicine, Louisiana State University, Baton Rouge, LA, United States; ^3^ Department of Virology, Immunology, and Microbiology, Boston University Chobanian & Avedisian School of Medicine, Boston, MA, United States; ^4^ Department of Pathology and Laboratory Medicine, Boston University Chobanian and Avedisian School of Medicine, Boston, MA, United States; ^5^ National Emerging Infectious Diseases Laboratories (NEIDL), Boston University, Boston, MA, United States; ^6^ Pennington Biomedical Research Center, Louisiana State University, Baton Rouge, LA, United States; ^7^ Department of Diagnostic Medicine and Pathobiology, College of Veterinary Medicine, Kansas State University, Manhattan, KS, United States

**Keywords:** SARS-CoV-2, COVID-19, respiratory infection, obesity, type 2 diabetes mellitus, T2DM, mouse model

## Abstract

Comorbidities, including obesity and type 2 diabetes mellitus (T2DM), are associated with increased disease severity and mortality following SARS-CoV-2 infection. Here, we investigated virus-host interactions under the effects of these comorbidities in diet-induced obesity (DIO) and leptin receptor-deficient (T2DM) mice following infection with SARS-CoV-2. DIO mice, as well as their lean counterparts, showed limited susceptibility to SARS-CoV-2 infection. In contrast, T2DM mice showed exacerbated pulmonary SARS-CoV-2 replication and delayed viral clearance associated with down-regulation of innate and adaptative immune gene signatures, ineffective type I interferon response, and delayed SARS-CoV-2-specific cell-mediated immune responses. While T2DM mice showed higher and prolonged SARS-CoV-2-specific immunoglobulin isotype responses compared to their lean counterparts, neutralizing antibody levels were equivalent. By silencing the leptin receptor *in vitro* using a human alveolar epithelial cell line, we observed an increase in SARS-CoV-2 replication and type I interferons. Altogether, our data provides for the first time evidence that disruption of leptin receptor signaling leading to obesity and T2DM induces altered type I interferon and cell-mediated responses against SARS-CoV-2, mediating increased viral replication and delayed clearance. These data shed light on the alteration of the innate immune pathway in the lung using in-depth transcriptomic analysis and on adaptive immune responses to SARS-CoV-2 under T2DM conditions. Finally, this study provides further insight into this risk factor aggravating SARS-CoV-2 infection and understanding the underlying cellular mechanisms that could help identify potential intervention points for this at-risk population.

## 1 Introduction

Severe Acute Respiratory Syndrome Coronavirus-2 (SARS-CoV-2) is responsible for the Coronavirus Disease 2019 (COVID-19) pandemic, associated with a respiratory disease of variable severity that may lead to the development of acute respiratory distress syndrome (ARDS) requiring intensive care and mechanical ventilation (Goyal et al., 2020; Tenforde et al., 2020; Wang et al., 2020). SARS-CoV-2 has a high transmissibility rate, and as of September 1^st^, 2024, the total number of confirmed infected patients has risen to 776 million people, with 7 million deaths (i.e., 0.91% mortality rate) (Johns Hopkins University & Medicine: Coronavirus Resource Center, 2022). Although the exact mechanism leading to acute respiratory distress syndrome (ARDS) following SARS-CoV-2 infection is not fully understood, the induction of a pulmonary “cytokine storm,” characterized by increased levels of inflammatory cytokines is considered to be one of the leading factors (Hojyo et al., 2020; Ragab et al., 2020; Hu et al., 2021). Additionally, dysregulation of type I interferon responses in the context of hyperinflammation in patients with severe COVID-19 has also been reported (Hadjadj et al., 2020; Ogger et al., 2022).

The global obesity epidemic is recognized as a significant public health issue (Meldrum et al., 2017; Temple, 2022). As of March 1^st^, 2024, an estimated 2.5 billion people are overweight, while 890 million are classified as obese worldwide (World Health Organization, 2024). Obesity has serious health consequences and is a high-risk factor for the development of type 2 diabetes mellitus (T2DM), hypertension, strokes, and various cancers (Pi-Sunyer, 2002). In the United States, obesity affects around 41.9% of the population, while T2DM has a prevalence of 11.6% (Centers for Disease Control and Prevention, 2024). Diabetes is a chronic disease characterized by hyperglycemia resulting from an impairment in insulin secretion and/or function. T2DM constitutes more than 95% of diabetes cases and is a result of insulin resistance coupled with β-cell insulin secretion dysfunction (DeFronzo et al., 2015). Obesity and T2DM have been identified as risk factors for increased severity of respiratory infections, such as Middle East Respiratory Syndrome (MERS)-coronavirus (Hui et al., 2018; Kulcsar et al., 2019) and influenza A virus (Morgan et al., 2010; O’Brien et al., 2012; Paich et al., 2013; Zhang et al., 2013; Cocoros et al., 2014; Ruiz et al., 2020). These are also recognized as important risk factors for hospitalization and the need for intensive care in COVID-19 patients (Altonen et al., 2020; Goyal et al., 2020; Moon et al., 2020; Richardson et al., 2020; Simonnet et al., 2020; Tamara and Tahapary, 2020; Tartof et al., 2020; Dennis et al., 2021; Gao et al., 2021; Hendren et al., 2021; Lv et al., 2022; Roy and Demmer, 2022). Patients suffering from these diseases are 3.40 times more likely to develop severe disease (Cai et al., 2020). Despite ongoing research on SARS-CoV-2 pathogenesis, the understanding of the specific effects of obesity and T2DM on SARS-CoV-2 infections and how these complex metabolic derangements precisely increase disease’s severity remain limited.

Leptin, a hormone primarily produced by adipocytes, regulates appetite, energy balance, and glucose metabolism. It promotes satiety by binding to the leptin receptor (LEPR) on hypothalamic neurons, activating the JAK-STAT pathway, leading to the phosphorylation of STAT3 (pSTAT3) which drives the production of anorexigenic peptides that suppress food intake and increase energy expenditure (Friedman, 2019; Mendoza-Herrera et al., 2021). Leptin also influences energy metabolism in peripheral tissues, such as lung epithelia and immune cells (predominantly macrophages and lymphocytes) (Malli et al., 2010; Kiernan and MacIver, 2021; Thieulent and Carossino, unpublished). In obese conditions, leptin resistance develops, impairing the body’s ability to regulate food intake despite elevated leptin levels, partly due to cytosolic LEPR inhibitors, such as Suppressor of Cytokine Signaling 3 (SOCS3) (Frederich et al., 1995; Enriori et al., 2007). Interestingly, both SOCS3 and STAT3 can negatively regulate type I interferon (IFN) responses, with the latter suppressing STAT1 through its sequestration (Ho and Ivashkiv, 2006; Carow and Rottenberg, 2014; Wang et al., 2019). The interaction between LEPR and type I IFN signaling is likely to shape responses to respiratory viral infections.

Understanding the molecular mechanisms behind the increased severity of SARS-CoV-2 infections in obese and T2DM patients is critical to understand the efficacy of vaccines and for the successful development of therapeutic strategies for this at-risk population. Here, we comparatively assessed the pathogenicity of SARS-CoV-2 in diet-induced obesity (DIO) and *Lepr*-deficient, T2DM mice, and further assessed virus-host interactions at the pulmonary level and adaptive cell- and humoral-mediated responses in the latter. Additionally, we correlated *in vivo* findings by establishing a link between leptin receptor silencing, excess leptin, and increased SARS-CoV-2 replication *in vitro*.

## 2 Materials and methods

### 2.1 Biosafety

All aspects of this study were approved by the Institutional Biosafety and Recombinant DNA Safety Committee, Office of Environmental Health and Safety at Louisiana State University (LSU) prior to the study initiation. Work with SARS-CoV-2 was performed in a biosafety level-3 (BSL-3 and ABSL-3) laboratory at the School of Veterinary Medicine, LSU by personnel equipped with powered air-purifying respirators.

### 2.2 Cells and viruses

African green monkey VERO C1008 cells [Vero 76, clone E6, Vero E6] (ATCC^®^ CRL-1587™, Manassas, VA) were maintained in Minimum Essential Medium (MEM) with Earl’s salts and L-glutamine (Corning, Corning, NY) supplemented with 10% fetal bovine serum (FBS; HyClone Laboratories, Inc., Logan, UT), 100 U/mL of Penicillin and 100 µg/mL Streptomycin (Gibco, Carlsbad, CA), and 0.25 µg/mL of Amphotericin B (Gibco). Human lung carcinoma cells (A549; NR-52268) and A549 expressing human angiotensin-converting enzyme 2 (A549-hACE2; NR-53821) were obtained from BEI Resources (Manassas, VA) and were maintained in Dulbecco’s Modified Eagle’s Medium (DMEM) (Gibco) supplemented with 10% FBS, 1% 1× non-essential amino acids (Gibco). A549-hACE2 cells were cultured in presence of 100 μg/mL of Blasticidin (Gibco).

The SARS-CoV-2 mouse-adapted MA10 strain (NR-55329) (Leist et al., 2020) and the SARS-CoV-2 recombinant infectious clone with enhanced Green Fluorescent Protein (ic-SARS-CoV-2-eGFP) (NR-54002) were obtained from BEI Resources and used after three and two passages on Vero E6 cells and A549-hACE2, respectively. Virus stocks were titrated by plaque assay and sequence-verified as previously described (Thieulent et al., 2023).

### 2.3 Mice

All mice included in this study were obtained from The Jackson Laboratory (JAX, Bar Harbor, ME) and were housed in groups of 3 to 4 per cage under a 12 h light:12 h dark cycle. Fourteen-week-old male C57BL/6J-60% diet-induced obese (DIO) mice (Strain #380050, B6 DIO; n=24) were used as a model of DIO (fed six weeks of standard chow diet [10% kcal from fat], followed by eight weeks of high-fat chow diet [60% kcal from fat]), and 14-week-old male C57BL/6J-10% mice (Strain #380056; B6 lean; n=24) were used as the respective control model (14 weeks of standard chow diet [10% kcal from fat]). B6 DIO mice had *ad libitum* access to water and a high-fat chow diet (60% kcal from fat, TD.06414, Envigo, Indianapolis, IN), whereas B6 lean mice had *ad libitum* access to water and standard chow diet (10% kcal from fat, LabDiet, St. Louis, MO). Eight-week-old male, *Lepr*-deficient, BKS.Cg-Dock7m +/+ Leprdb/J mice (Strain #000642, BKS db/db; n=32) were used as a model of T2DM (Coleman, 1978; Chen et al., 1996) while the wildtype control corresponded to the lean counterparts (BKS.Cg-Dock7m +/+ Leprdb/J wildtype control (Strain #000642; BKS +/+; n=32)) were used as lean control. Both BKS db/db (*Lepr*-deficient, T2DM) and BKS +/+ (lean) mice had *ad libitum* access to water and a standard chow diet (10% kcal from fat, LabDiet). As DIO can only be developed in male mice (Oraha et al., 2022), only male mice were used in this study to compare the two models.

### 2.4 Experimental design and intranasal inoculation

Two separate experiments were conducted in this study. In the first experiment (obesity study), B6 DIO (n=9) and B6 lean mice (n=9) were intranasally infected with the SARS-CoV-2 MA10 strain as previously described (Thieulent et al., 2023). In the second experiment (T2DM study), *Lepr*-deficient, T2DM (n=24) and lean mice (n=24) were similarly infected. Infected DIO and DIO control mice (n=3 per group) were euthanized at 2 dpi, 4 dpi, and 7 dpi. Infected *Lepr*-deficient, T2DM, and lean (n=4 per group) were euthanized at 2 dpi, 4 dpi, 7 dpi, 10 dpi, 14 dpi, and 21 dpi. For each study, mock-infected mice were included and euthanized at 7 dpi and 21 dpi.

### 2.5 Blood glucose measurement

Blood glucose was measured for each mouse 2 to 4 days prior to experimental infection and at the time of euthanasia using OneTouch Verio^®^ Test Strips (LifeScan, Malvern, PA) and OneTouch Verio^®^ Flex Blood Glucose Meter (LifeScan). All mice were fasted 8 hours before blood glucose measurement.

### 2.6 Clinical monitoring and euthanasia

An IACUC-approved clinical scoring system was utilized to monitor disease progression as previously described (Carossino et al., 2022; Thieulent et al., 2023). Clinical signs and body weight were monitored twice per day until 4 dpi, and then once a day for the duration of the experiment. Euthanasia was performed via isoflurane overdose and subsequent cervical dislocation following the AVMA Guidelines for the Euthanasia of Animals (American Veterinary Medical Association, 2020).

### 2.7 Tissue collection, viral RNA quantitation by RT-qPCR, and virus titer determination by plaque assay

Lung and brain tissues were collected and processed for plaque assay and viral RNA isolation for quantification by SARS-CoV-2 ORF1ab-specific RT-qPCR as previously described (Thieulent et al., 2023). Viral titers (PFU) and viral RNA genomic copies were determined per milligram of tissue.

### 2.8 Histopathology and SARS-CoV-2-specific immunohistochemistry

Following fixation, the entire head was decalcified in Immunocal™ Decalcifier (StatLab, McKinney, TX) for 5 days followed by thorough rinsing in tap water per manufacturer’s recommendations before performing a mid-sagittal section. Tissues (lung and heads) were subsequently processed, embedded in paraffin, and four-micron sections stained with hematoxylin and eosin following standard histological procedures. SARS-CoV-2 N-specific immunohistochemistry (IHC) was performed as previously described (Carossino et al., 2020; Thieulent et al., 2023). Histological alterations and SARS-CoV-2 antigen abundance were semi-quantitatively scored as previously described (Thieulent et al., 2023).

### 2.9 RNAscope^®^ *in situ* hybridization and signal quantification

The antisense probe targeting murine *Ifnb1* mRNA was purchased from Advanced Cell Diagnostics (ACD, cat. No. 406538, Newark, CA). The RNAscope^®^ ISH assay was performed using the RNAscope^®^ 2.5 LSx reagent kit (Advanced Cell Diagnostics) on the automated BOND RX^m^ platform (Leica Biosystems, Buffalo Grove, IL) as described previously (Thieulent et al., 2023). RNAscope^®^ ISH slides were scanned at 40X magnification using a NanoZoomer HT whole slide scanner (Hamamatsu, Japan). Quantitative analysis was performed using the ISH module available through HALO (IndicaLabs, Albuquerque, NM) digital pathology image analysis software. The slides were manually annotated to only include pulmonary parenchyma in the analysis, and the number of positive cells/mm2 of lung tissue was determined following analysis of whole slide images.

### 2.10 Multiplex fluorescent immunohistochemistry and quantitative image analysis.

Two mfIHC panels (panel 1: SARS-CoV-2 N, CXCL10 and pSTAT1; panel 2: pSTAT1, pSTAT3, and pSTAT5) were developed as previously described (Carossino et al., 2022) (**Supplementary Material 1**) and using the antibodies indicated in **Supplementary Table S1**. Subsequently, whole-slide scans were obtained using a PhenoImager Quantitative Pathology Imaging System (Akoya Biosciences, Boston, MA), and digitized whole-slide scans were analyzed using the image analysis software HALO (Indica Labs, Inc.). Exposures for all Opal dyes on the Vectra were set based on regions of interest with strong signal intensities to minimize exposure times and maximize the specificity of the signal detected. Images were unmixed using spectral libraries affiliated with each respective Opal fluorophore including removal of autofluorescence. Slides were manually annotated to include only lung parenchyma. Visualization threshold values were adjusted in viewer settings to reduce background signals. Area quantification (AQ) was performed to determine the number of positive cells for each marker per mm^2^ of tissue.

### 2.11 Total cellular RNA isolation and gene expression analysis for selected cytokines/chemokines

Gene expression analysis of cellular genes was measured as previously described (Thieulent et al., 2023). For the A549-hACE2 cells, the supernatant was removed and 350 µL of Buffer RLT Plus containing 1% of β-mercaptoethanol was added to the cell monolayer, cells were scraped using pipet tips, and the cell lysate was collected for RNA extraction. Specific forward and reverse primers for genes of interest were designed using the Primer-BLAST website (NCBI, NIH) and are shown in **Supplementary Table S2**. Reactions were performed in duplicate and expression of genes of interest were normalized to three stable housekeeping genes (*Actb, Gusb*, and *Rpl13b* for mouse genes; *GAPDH, GUSB*, and *RPL13A* for human genes) as previously described (Yin et al., 2010; Thieulent et al., 2023).

### 2.12 Bulk mRNA sequencing

Total RNA was extracted from ≈30 mg of lung tissues previously frozen at -80°C in RNAlater™ stabilization solution (ThermoFisher Scientific) using the RNeasy Plus Mini Kit (QIAGEN) according to the manufacturer’s recommendations. RNA concentration and purity were assessed using NanoDrop™ 2000 spectrophotometer (ThermoFisher Scientific). Pure RNA extracts (2.0 ≥ A260/A280 ratio ≤ 2.2) were sent to the Genomics Core Facility, Pennington Biomedical Research Center (Baton Rouge, LA) for sequencing. Sequencing libraries were constructed using QuantSeq 3’ mRNA-Seq Library Prep Kit FWD (Lexogen, Vienna, Austria) according to the manufacturer’s recommendations. Briefly, cDNA libraries were generated from mRNA using oligo(dT) primers. Then RNA was removed, and a second strand cDNA synthesis was initiated using random primers. All the primers used contained a partial Illumina-compatible linker sequence. After amplification, the libraries were sequenced using Illumina NextSeq 2000 System (Illumina, San Diego, CA) following the manufacturer’s recommendations, then analyzed on the Bioanalyzer High Sensitivity DNA chip (Agilent, Santa Clara, CA) to verify the correct library size. The libraries were sequenced on the Illumina NextSeq 2000 System (Illumina, San Diego, CA) at 75bp forward read and 6bp forward index read.

### 2.13 Bioinformatic analysis of RNAseq data

Raw sequencing files were processed using Lexogen Quantseq pipeline V1.8.8 on the Bluebee platform for quality control and mapping (genome: mouse/GRCm38). The quality assessment of raw and trimmed reads was carried out using FastQC. Trimming of the adapter content and quality trimming was performed using Cutadapt (a phred score ≤ 20). Differentially expressed genes (DEGs; down-regulated genes cutoff: Log_2_FC <0.5, up-regulated genes cutoff: Log_2_FC >0.5) of each condition combination (lean vs *Lepr*-deficient, T2DM mice; mock, 2 dpi and 4 dpi) was assessed in R environment (version 4.2.1) using DESeq2 Bioconductor package (version 1.36.0; FDR < 0.1) (Love et al., 2014). Principal component analysis was performed using ggplot2 R package (Wickham, 2016). The list of over-represented and under-represented Gene ontology (GO) terms of differentially expressed genes was obtained using PANTHER Classification System (pantherdb.org) and REVIGO (revigo.orb.hr) websites as previously described (Bonnot et al., 2019). GO terms with a fold enrichment > 1 were graphically represented using R with ggplot2 package. Gene set enrichment analysis (GSEA) analysis and visualization were performed using GSEA, Cytoscape and EnrichmentMap as previously described (Subramanian et al., 2005; Reimand et al., 2019). Gene sets with a normalized enrichment score (NES) were represented. UpSet plot was created using R with UpSetR package from DEGs with a log2 fold change (log2FC) > 0.5 (Conway et al., 2017). DEGs were submitted to Ingenuity Pathway Analysis (IPA; QIAGEN Inc., https://www.qiagenbioinformatics.com/products/ingenuitypathway-analysis) for biological process analysis using a False Discovery Rate (FDR) cut-off of 0.1. Pathways with a Z-score ≥ 2 were considered significantly upregulated, whereas pathways with a Z-score ≤ 2 were considered significantly downregulated (Krämer et al., 2014). Pathways that were statistically enriched were exported and plotted using R with pheatmap package. Proteins interaction network was generated on STRING (https://www.string-db.org) using the lists of the up-regulated DEGs with *Mus musculus* selected as organism. Network was then exported to Cytoscape (version 3.9.1) (Shannon et al., 2003) and the top 15 nodes (hub genes) ranked by degree were identified and represented using Cytoscape plugin CytoHubba (version 0.1) (Wu et al., 2020).

### 2.14 *Ex vivo* splenocyte stimulation with SARS-CoV-2 peptides

Spleens were collected and placed into a 2 mL sterile tube containing 1 mL of complete RPMI medium (Gibco) and subsequently transferred into a 35 mm Petri dish containing 2 mL of complete RPMI medium. Splenocytes were extracted by incision of the splenic capsule and extruding cells out. Splenocytes were filtered through a 70 µm cell strainer into a 50 mL conical tube. Splenocytes were centrifuged at 300 × g for 10 min at 4°C. After removing the supernatant, cells were resuspended in 5 mL of RBC Lysis Buffer (eBioscience, San Diego, CA) and incubated at RT for 5 min with occasional shaking. The reaction was stopped by adding 25 mL of 1X PBS (Gibco). Splenocytes were centrifuged at 300 × g for 10 min at 4°C and resuspended in complete RPMI medium. A total of 1 × 10^6^ cells were transferred into 24-well plates. Splenocytes were stimulated with a mixture of SARS-CoV-2 S-specific (PepTivator^®^ SARS-CoV-2 Prot_S Complete [Miltenyi Biotech: 130-127-951], targeting the complete protein coding sequence [aa 5-1273; GenBank MN908947.3, UniProt: QHD43416.1]), SARS-CoV-2 N-specific (PepTivator^®^ SARS-CoV-2 Prot_N [Miltenyi Biotech: 130-126-698], targeting the complete sequence protein coding sequence [GenBank MN908947.3, UniProt: QHD43423.2]), or control peptides (PepTivator^®^ Negative Control [Miltenyi Biotech: 130-131-610]) at a concentration of 0.6 nmol/mL, and in presence of 5 μg/mL of Brefeldin A (BioLegend, San Diego, CA) for 5 h at 37°C with 5% CO_2_. Stimulation with a mixture of 20ng/mL of phorbol-12-myristate-13-acetate (PMA; ThermoFisher Scientific) and 1 µg/ml of ionomycin (ThermoFisher Scientific) was used as a positive control. Splenocytes were collected and washed with Fluorescence-activated cell sorting (FACS) buffer (1X PBS containing 0.1% sodium azide (VWR, Radnor, PA) and 1% heat-inactivated sterile-filtered goat serum (EquiTech-Bio, Inc., Kerrville, TX) and labelled for 30 min at 4°C with the following cell surface markers: PE-conjugated rat anti-mouse CD8b clone H35-17.2 (BD Pharmingen, Franklin Lakes, NJ; diluted 1:200 in FACS buffer) and APC-conjugated rat anti-mouse CD4 clone RM4-5 (BS Pharmingen; diluted 1:200 in FACS buffer), or appropriate isotype controls, PE-conjugated rat IgG2b κ isotype control clone A95.1 (BD Pharmingen) and APC-conjugated rat IgG2b, κ isotype clone R35-95 (BD Pharmingen), respectively. Splenocytes were fixed/permeabilized with BD Cytofix/Cytoperm solution (BS Biosciences) for 20 min at 4°C and labeled for 30 min at 4°C with Alexa Fluor^®^ 488-conjugated anti-mouse IFNγ antibody (Biolegend; diluted 1:200 in BD Perm/Wash™ buffer) (BD Biosciences) and the appropriate isotype control Alexa Fluor^®^ 488-conjugated rat IgG1, κ Isotype control antibody (Biolegend). Flow cytometry data was collected using a BD FACS Calibur™ cytometer with 10^5^ events and analyzed with FlowJo version 10 software (BD Biosciences).

### 2.15 Virus neutralization test

Neutralizing antibodies were determined using a standard virus neutralization test (VNT). Briefly, 1.5 × 10^4^ Vero E6 cells/well were plated into a 96-well plate and incubated at 37°C for 24h. On the next day, sera were decomplemented at 56°C for 30 min, and two-fold serial dilutions of each serum (1:4 to 1:2,048) were performed in duplicate on 96-well plates using complete MEM. A total of 25 µl of diluted sera were mixed with an equivalent volume of a working dilution of SARS-CoV-2 MA10 strain containing 100 median Tissue Culture Infectious Dose (TCID_50_) and incubated for 1 h at 37°C. The serum/virus mixture was then added to the Vero E6 cells containing 100 µl of fresh complete MEM. After 72 h at 37°C, the neutralizing antibody titer was recorded as the reciprocal of the highest serum dilution that provided at least 50% protection against the reference virus. Pooled sera from vaccinated individuals with BNT162b2 Pfizer vaccine (BEI Resources, NRH-17727) and normal mouse serum (Jackson ImmunoResearch, West Grove, PA) were used as positive and negative controls, respectively.

### 2.16 SARS-CoV-2 specific immunoglobulin isotyping by indirect ELISA

Indirect ELISA for isotyping specific antibodies against the recombinant nucleoprotein (N) and spike receptor-binding domain (S-RBD) of the SARS-CoV-2 USA-WA1/2020 strain were performed as previously described (Gaudreault et al., 2020). High-binding 96-well plates (Greiner, Kremsmünster, Austria) were coated overnight at 4°C with 100 ng of SARS-CoV-2 N or S-RBD antigens diluted in 100 μL of carbonate-bicarbonate buffer (Sigma-Aldrich, St. Louis, MO). The following day, the plates were washed twice with 1X PBS and blocked with 200 μL of casein blocking buffer (Sigma-Aldrich) for 1h at RT. The plates were washed three times with 1X PBS-Tween 20 (Bio-Rad, Hercules, CA) (0.5% Tween 20 in 1X PBS). Serum samples were heat-inactivated for 30 min at 56°C and pre-diluted at 1:400 in casein blocking buffer. 100 μL of pre-diluted sera were added in duplicate in the ELISA plates and incubated for 1h at RT. The plates were washed three times with 1X PBS-Tween-20 and 100 μL of secondary antibodies (Goat Anti-Mouse IgM-HRP (SouthernBiotech, Birmingham, AL; 1021-05), Goat Anti-Mouse IgG, Human ads-HRP (SouthernBiotech; 1030-05), Goat Anti-Mouse IgG1-HRP (SouthernBiotech; 1071-05), Goat Anti-Mouse IgG2a-HRP (SouthernBiotech; 1081-05), Goat Anti-Mouse IgG2b-HRP (SouthernBiotech; 1091-05) and Goat Anti-Mouse IgG2c-HRP (SouthernBiotech; 1078-05)), pre-diluted at 1:2500 in 1X PBS, were added to each well and incubated for 1h at RT. The plates were washed five times with 1X PBS-Tween-20 and 100 μL of TMB Substrate Set (Biolegend) was added to each well. The plates were incubated for 5 min at RT in the dark and 100 μL of Stop Solution for TMB Substrate (Biolegend) was added to each well. The OD_450nm/570nm_ ratio of each ELISA plate was subsequently read on a Spark^®^ multimode microplate reader (Tecan Life Technology, Männedorf, Switzerland). The cut-off for a sample being called positive was determined as follows: Average OD_450nm/570nm_ of negative serum + 3 × standard deviation.

### 2.17 Leptin receptor silencing and recombinant leptin treatment and SARS-CoV-2 infection of A549-hACE2 cells

A total of 1 × 10^5^ A549-hACE2 cells were seeded on 24-well plates and incubated at 37°C for 24h in the presence of 100 μg/mL of Blasticidin. The following day, the medium was replaced with 450 μL of Blasticidin-free medium, and the cells were transfected with siRNAs targeting *LEPR* (Invitrogen: s224011) and negative control (Silencer™ Select Negative Control No. 1 siRNA, Invitrogen). Lipofectamine^®^ RNAiMAX Reagents (Invitrogen) were used for transfection following the manufacturer’s recommendations. Twenty-four hours post-transfection (hpt), recombinant human leptin (rLeptin; R&D Systems, Minneapolis, MN) was added to the cell supernatant to reach final concentrations of 0 ng/mL, 10 ng/mL, and 100 ng/mL. At 48 hpt, the medium was removed, and cells were infected with 1 × 10^4^ PFU of ic-SARS-CoV-2-eGFP. After 1h of adsorption at 37°C, virus inoculum was removed and medium containing 0 ng/mL, 10 ng/mL, or 100 ng/mL of rLeptin was added. At 12 hpi, 24 hpi, and 48 hpi, cells were harvested with Trypsin-EDTA (0.05%; Gibco), pelleted by centrifugation at 500 × g for 10 min, and fixed with 4% paraformaldehyde (PFA) for 1h at room temperature. Cells were finally rinsed three times in FACS buffer before analysis on a BD FACS Calibur™ cytometer (5 × 10^4^ events). Data were analyzed with FlowJo version 10 software (BD Biosciences). Experiments were performed in triplicate wells and repeated in three independent experiments.

### 2.18 Statistics

Statistical analysis and graphics were performed using GraphPad Prism v10.1.1 statistical analysis software (GraphPad, San Diego, CA). Initial weights and blood glucose concentration were analyzed using unpaired t-test. Histological, antigen scores and virus neutralization titers were analyzed using multiple Mann-Whitney tests. Viral loads (genomic copies/mg and pfu/mg) were analyzed using two-way ANOVA with Sidak *post-hoc* test. Relative gene expression levels, immunoglobulin levels, and flow cytometry data were analyzed using two-way ANOVA with Tukey *post-hoc* test. *P*-values have been adjusted to account for multiple comparisons for Mann-Whitney tests and two-way ANOVA. Data are expressed as mean ± standard deviation (SD). Statistical significance was set at *P* ≤ 0.05. Statistical significances on Figures are labeled as follows: * *P* ≤ 0.05, ** *P* ≤ 0.01, *** *P* ≤ 0.001.

## 3 Results

### 3.1 SARS-CoV-2-infected obese mice do not exhibit increased morbidity or increased viral replication

To evaluate the effect of obesity on SARS-CoV-2 infection, 14-week-old male C57BL/6J-60% diet-induced obese (DIO) mice and age-matched male C57BL/6J-10% (lean) mice were intranasally infected with a sub-lethal dose of 2 x 10^4^ PFU of SARS-CoV-2 mouse-adapted MA10 strain and monitored for 7 days following infection (**Supplementary Figure S1A**). At the time of infection, the weight of the B6 DIO mice (36.55 ± 3.74 g) was significantly higher than that of the B6 lean group (29.15 ± 1.78 g) (*P* ≤ 0.001; **Supplementary Figure S1B**). However, no significant difference in glycemia was noted between B6 DIO mice (233 ± 53.87 mg/dl) and B6 lean mice (228.50 ± 37.76 mg/dl) (*P* = 0.815; **Supplementary Figure S1C**), demonstrating that 14-week-old B6 DIO mice remained able to control the glycemia. No weight loss, clinical signs, lethality, or changes in blood glucose were observed during the 7 days post-infection (**Supplementary Figures S1D, S1E**). Furthermore, both B6 lean and B6 DIO mice exhibited a low level of infectious virus, viral RNA, viral antigen, and limited pulmonary histological alterations, with no differences observed between the two groups (**Supplementary Figures S2A–S2T**). There was also no evidence of neuro-invasion (**Supplementary Figures S3A–S3D**). These findings suggest that B6 mice have low susceptibility to SARS-CoV-2 MA10 strain infection, as previously reported (Leist et al., 2020; Thieulent et al., 2023), and DIO did not increase their susceptibility.

### 3.2 SARS-CoV-2 induces exacerbated viral replication and prolonged infection in *Lepr*-deficient, T2DM mice

**Figure 1:**
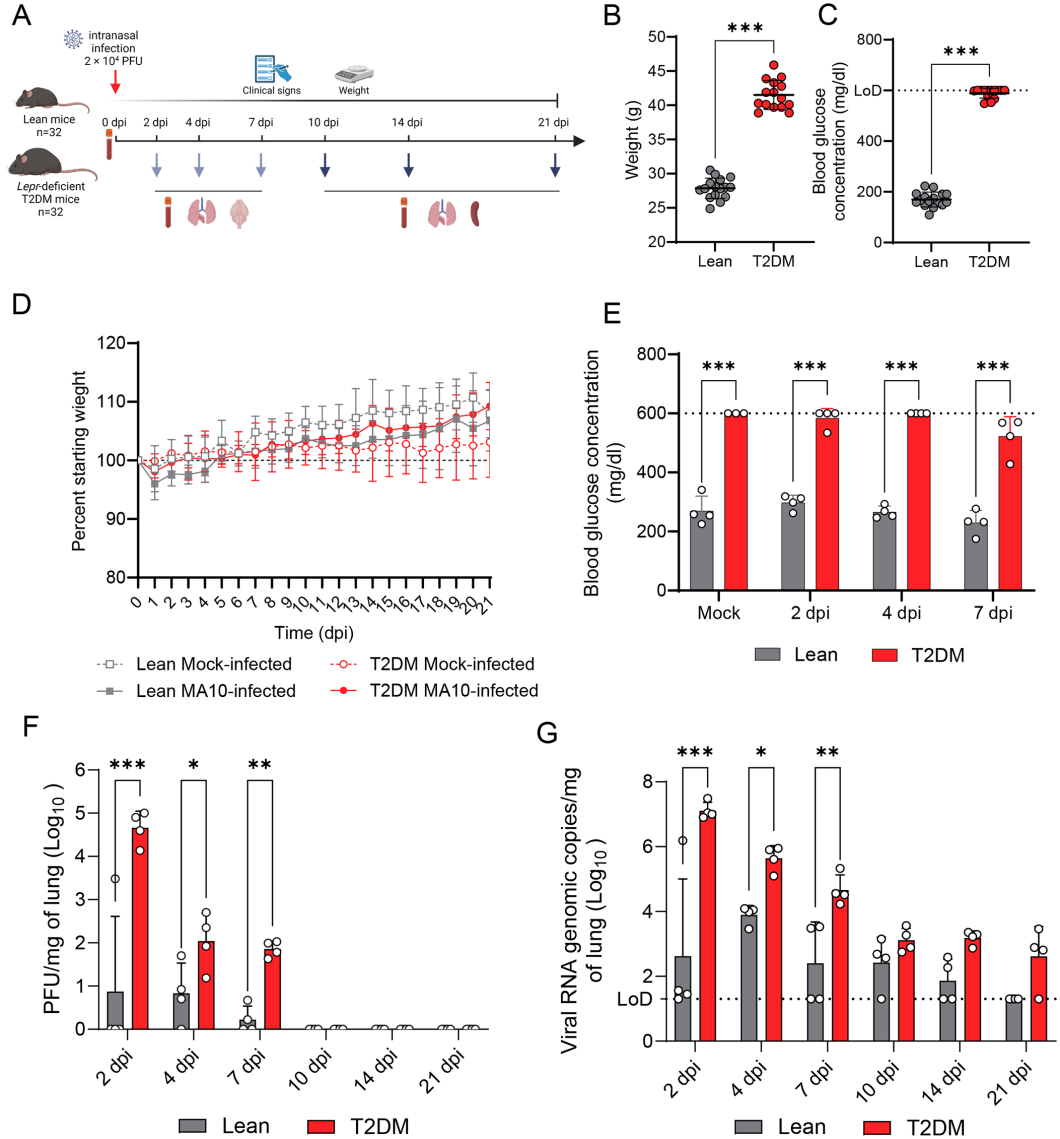
*Lepr*-deficient, T2DM mice exhibit exacerbated viral replication following SARS-CoV-2 MA10 infection. **(A)** Experimental approach for evaluating SARS-CoV-2 infection in *Lepr*-deficient, T2DM mice. Lean (n=24) and *Lepr*-deficient, T2DM (n=24) mice were intranasally infected with 2 × 10^4^ PFU of SARS-CoV-2 MA10 strain, or mock-infected (n=4) and monitored up to 21 dpi. Weight and blood glycemia were monitored. **(B)** Weight difference between *Lepr*-deficient, T2DM, and lean mice before infection. **(C)** Glucose concentration measured in the blood of fasted *Lepr*-deficient, T2DM, and lean mice before infection. The dotted line represents the upper limit of detection (LoD = 600 mg/dl). **(D)** Body weight change of mock-infected or SARS-CoV-2-infected *Lepr*-deficient, T2DM, and lean mice. **(E)** Glucose concentration measured in the blood of fasted mock-infected and SARS-CoV-2 infected *Lepr*-deficient, T2DM, and lean mice at 2 dpi, 4 dpi, and 7 dpi. **(F)** Infectious viral particles quantified in the lung of infected *Lepr*-deficient, T2DM and lean mice (n=4 per time point). **(G)** Viral RNA quantified in the lungs of infected *Lepr*-deficient, T2DM and lean mice (n=4 per time point). Bars represent the mean ± standard deviation. Dotted line represents the limit of detection (LoD). *P ≤ 0.05; **P ≤ 0.01; ***P ≤ 0.001.

The effect of T2DM on SARS-CoV-2 infection was evaluated by intranasal inoculation of 12-week-old male *Lepr*-deficient, T2DM (BKS db/db), and 12-week-old male wildtype control (BKS +/+, lean) mice as described above, and monitored up to 21 dpi (**Figure 1A**). *Lepr*-deficient, T2DM mice are obese (mean body weight of 41.50 ± 2.12 g) compared to their lean counterparts (mean body weight 27.85 ± 1.49 g) (*P* ≤ 0.001; **Figure 1B**) and hyperglycemic, with glycemia levels >3-fold higher than lean counterparts (*P* ≤ 0.001; **Figure 1C**). Following SARS-CoV-2 infection, no significant weight loss, clinical signs of disease, lethality, or changes to glycemia were recorded during the 21 days following infection (**Figures 1D, E**). Infectious viral titers and viral RNA genomic copies in the lung were significantly higher in *Lepr*-deficient, T2DM mice compared to lean mice between 2 and 7 dpi (*P* values ≤ 0.05; **Figures 1F, G**). Additionally, while the infectious virus was recovered from all SARS-CoV-2-infected *Lepr*-deficient, T2DM mice, infectious virus was only detected in 1/4, 3/4, and 2/4 lean mice at 2 dpi, 4 dpi, and 7 dpi, respectively. (**Figure 1G**). Viral clearance occurred earlier in lean mice compared to *Lepr*-deficient, T2DM mice, with viral titers of <10 PFU/mg detected in only 2/4 lean mice at 7 dpi, while infectious virus was detected in all *Lepr*-deficient, T2DM mice at this timepoint (average: 77 ± 31 PFU/mg). No infectious virus was identified in the lungs of both groups of mice from 10 dpi to the end of the study (21 dpi). Although no significant differences were observed at 10, 14 and 21 dpi, RT-qPCR data supports the trend toward delayed viral clearance in *Lepr*-deficient, T2DM mice. In lean mice, viral genomic RNA levels gradually decreased in the lungs at 10 dpi (3/4), 14 dpi (2/4), and 21 dpi (0/4). In contrast, genomic RNA persisted in the lungs of all *Lepr*-deficient, T2DM mice at 10 dpi, 14 dpi, and in 3/4 mice at 21 dpi.

**Figure 2:**
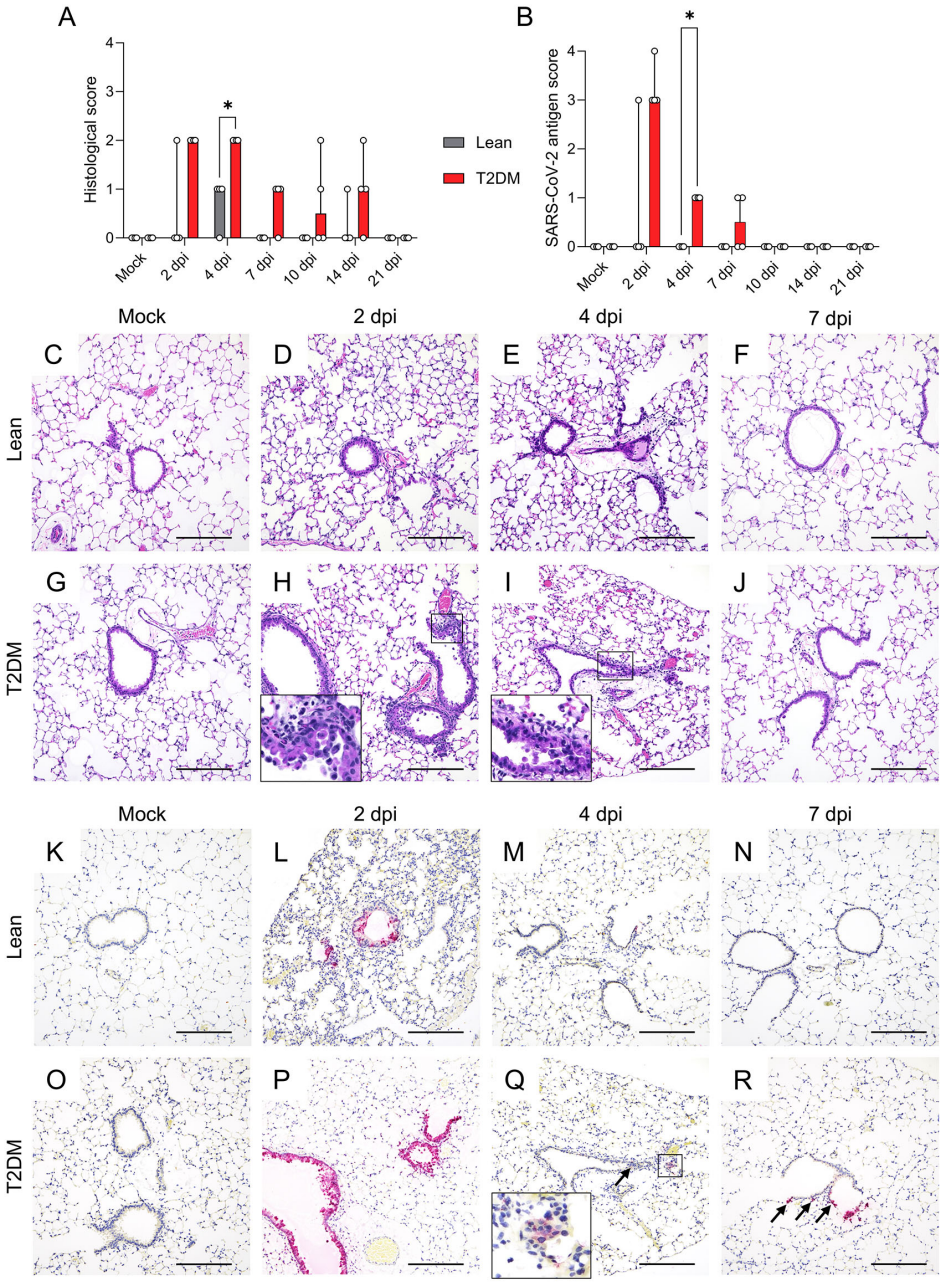
Comparative temporal analysis of SARS-CoV-2 MA10 replication and pathological alterations in the lung of *Lepr*-deficient, T2DM, and lean mice. **(A)** Histological scores and **(B)** SARS-CoV-2 antigen score in the lung of *Lepr*-deficient, T2DM, and lean mice (n=4 per time point). Bars represent the median ± range. Temporal histologic lesions (H&E) **(C–J)** and viral antigen (Fast Red) abundance and distribution **(K–R)** in the lung of T2DM and lean mice. *Lepr*-deficient, T2DM mice exhibit bronchiolar epithelial cell necrosis and peribronchiolar inflammation (necrotizing bronchiolitis) through 4 dpi (**H** and **I**, insets). Minimal mononuclear infiltrates are noted. No significant histologic changes were noted in the lungs of lean mice **(D–F)**. Abundant viral antigen within bronchiolar and, rarely, alveolar epithelial cells was noted in *Lepr*-deficient, T2DM mice through 7 dpi compared to lean mice **(P–R)** compared to **(L–N)**. Arrows indicate viral antigen in the cytoplasm of scattered alveolar epithelial cells. × 200 total magnification (Scale bar: 200 μm). *P ≤ 0.05.

Correlated with viral titers and RNA genomic copies, lean mice had minimal necrotizing bronchiolitis with epithelial viral antigen at 2 dpi (1/4 animals) to mild peribronchiolar lymphocytic inflammation but repaired bronchiolar epithelium and absence of viral antigen at 4 dpi (3/4 animals) (**Figures 2A–F, K–N**). No histologic lesion or viral antigen were detected in lean mice at 10, 14, and 21 dpi (**Supplementary Figures S4A–S4P**). In contrast, *Lepr*-deficient, T2DM mice exhibited mild multifocal necrotizing bronchiolitis extending up to 4 dpi, followed by only minimal multifocal interstitial pneumonia characterized by few peribronchiolar, perivascular and interstitial lymphocytes and macrophages that persisted through 14 dpi (**Figures 2A, G–J**, and **Supplementary Figure S4**). Histological changes and viral titers in the lungs of *Lepr*-deficient, T2DM mice were associated with significantly high levels of SARS-CoV-2 N antigen in bronchiolar epithelial cells and in rare alveolar epithelial cells (alveolar type 1 cells) at 2 dpi and with lower levels persisting through 7 dpi (**Figures 2B, O–R**). Angiotensin-converting enzyme 2 (ACE2) and the transmembrane serine protease 2 (TMPRSS2) play a crucial role in SARS-CoV-2 binding and entry in the cells (Matsuyama et al., 2010; Hoffmann et al., 2020). Here, a significant reduction of both *Ace2 (P* = 0.008) and *Tmprss2* (*P* = 0.047) mRNA expression was measured in the lung of *Lepr*-deficient, T2DM at 2 dpi, likely associated with bronchial epithelial turnover (**Supplementary Figures S5A, S5B**) as previously suggested (Thieulent et al., 2023). The lack of neuro-invasion was verified by RT-qPCR, infectious virus titration, viral antigen detection, and histological examination. (**Supplementary Figures S3E–S3H**). Overall, these data demonstrated that the BKS lean mice were able to successfully control SARS-CoV-2 infection within the initial 4 dpi, whereas *Lepr*-deficient, T2DM mice showed exacerbated SARS-CoV-2 replication, delayed clearance, and persisting viral antigen and histologic lesions in the lungs.

### 3.3 Global transcriptome analysis of non-infected *Lepr*-deficient, T2DM mice reveals baseline downregulation of key pulmonary immune pathways

**Figure 3:**
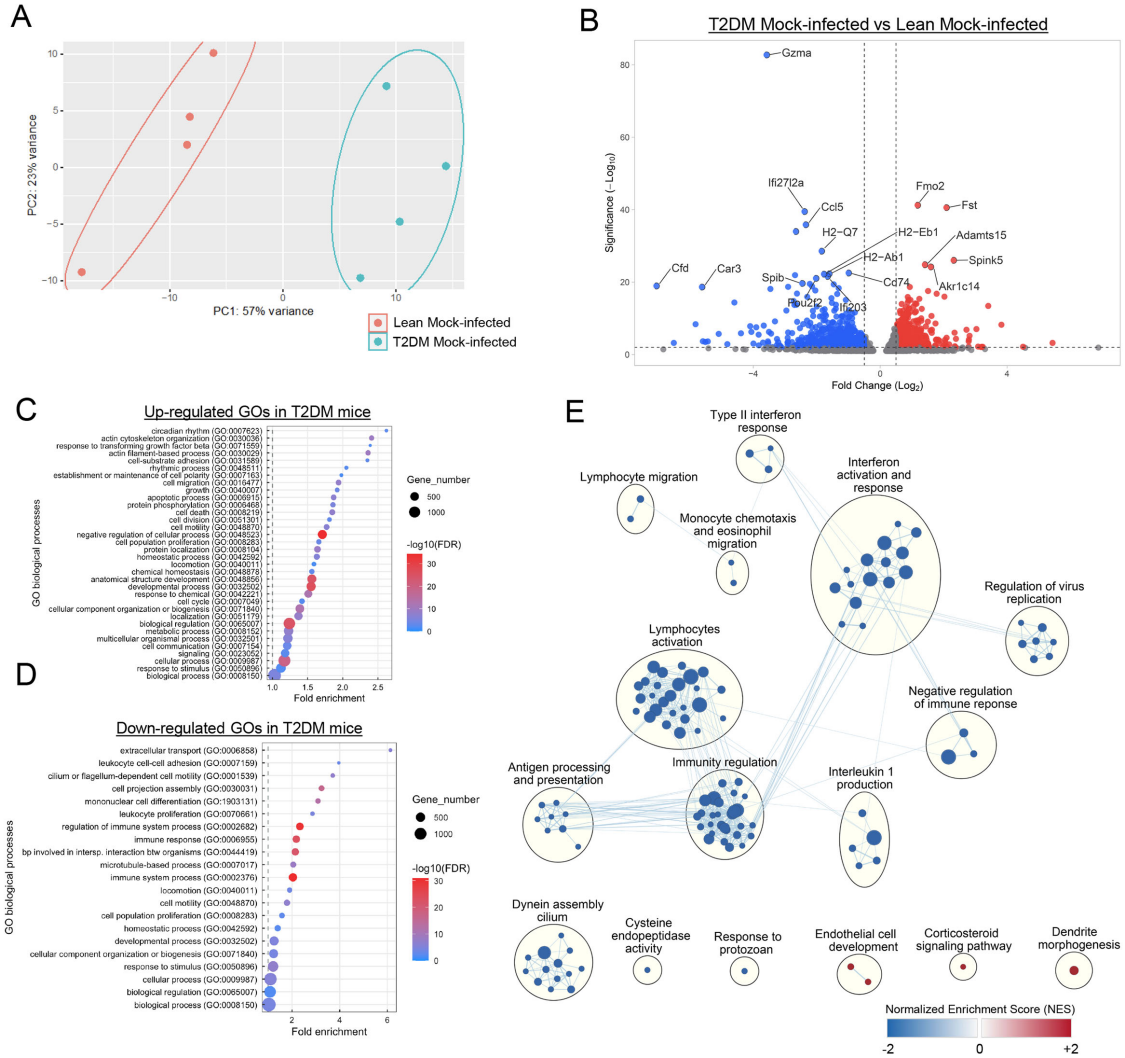
Baseline downregulation of genes related to immune response in the lung of *Lepr*-deficient, T2DM mice. **(A)** Principal component analysis (PCA) shows a clear separation between the differentially expressed genes (DEGs) in the lung of mock-infected lean mice (n=4) and the lung of mock-infected *Lepr*-deficient, T2DM mice (n=4). Each dot indicates a single sample colored by group: lean blue; T2DM: red. **(B)** Volcano plot showing the significantly down-regulated genes (blue) and up-regulated genes (red) in the lungs of *Lepr*-deficient, T2DM mice when compared to the lungs of lean mice. Cutoffs: Fold Change (Log*2*): 0.5, Significance (-Log_10_): 2. **(C)** Up-regulated and **(D)** down-regulated gene ontology (GO) biological processes observed in the lungs of mock-infected *Lepr*-deficient, T2DM mice when compared to the mock-infected lean mice. Most of the up-regulated GO biological processes are related to cell structure while most of the down-regulated GO biological processes are related to immune response and cell motility **(E)** Enrichment map representing the down-regulated (blue) and up-regulated (red) genes sets in the lung of mock-infected *Lepr*-deficient, T2DM mice when compared to the mock-infected lean mice. Each node represents a gene set, and edges (blue lines) represent the number of genes overlapping between two gene sets. Clusters of nodes were labeled using the AutoAnnotate Cytoscape application. Most gene sets in the lungs of *Lepr*-deficient, T2DM mice were downregulated. These sets were associated with immune regulation, lymphocyte activation, interferon activation and response, type II interferon response, and the regulation of viral replication, among other functions.

We performed bulk RNAseq and global transcriptome analysis of the pulmonary parenchyma derived from mock-infected *Lepr*-deficient, T2DM, and lean mice to assess transcriptional signatures at baseline that would suggest increased susceptibility to viral infection. Basic gene expression profile comparison by principal component analysis (PCA) revealed distinct clustering between gene signatures in *Lepr*-deficient, T2DM, and lean mock-infected mice (**Figure 3A**). A total of 2,139 differentially expressed genes (DEGs) were identified between the lungs of *Lepr*-deficient, T2DM, and lean mice, including 767 up-regulated DEGs (log_2_ FC > 0.5) and 1,372 down-regulated DEGs (log_2_ FC < 0.5) in the lung of the former group (**Figure 3B**). Identification of the most representative up-regulated Gene Ontology (GO) terms in the lung of *Lepr*-deficient, T2DM mice demonstrated that they were mainly associated with cell structure and cell development (**Figure 3C**), while the most down-regulated GO terms revealed a decrease in immune response-related processes (**Figure 3D**). Gene Set Enrichment Analysis (GSEA) was subsequently performed, and results demonstrated that most of the gene sets downregulated in the lung of *Lepr*-deficient, T2DM mice were related to immunity regulation, lymphocyte activation, interferon activation and response, type II interferon response, regulation of viral replication, among others (**Figure 3E**). Overall, these results demonstrate that, at baseline, the global transcriptional signature in *Lepr*-deficient, T2DM mice reveals a significant downregulation of key local immune processes that are critical for control of viral replication, and that could suggest increased susceptibility to SARS-CoV-2 infection.

### 3.4 *Lepr*-deficient, T2DM mice show heightened interferon beta and inflammatory cytokine response associated with STAT1 activation but fail to control early SARS-CoV-2 replication

**Figure 4:**
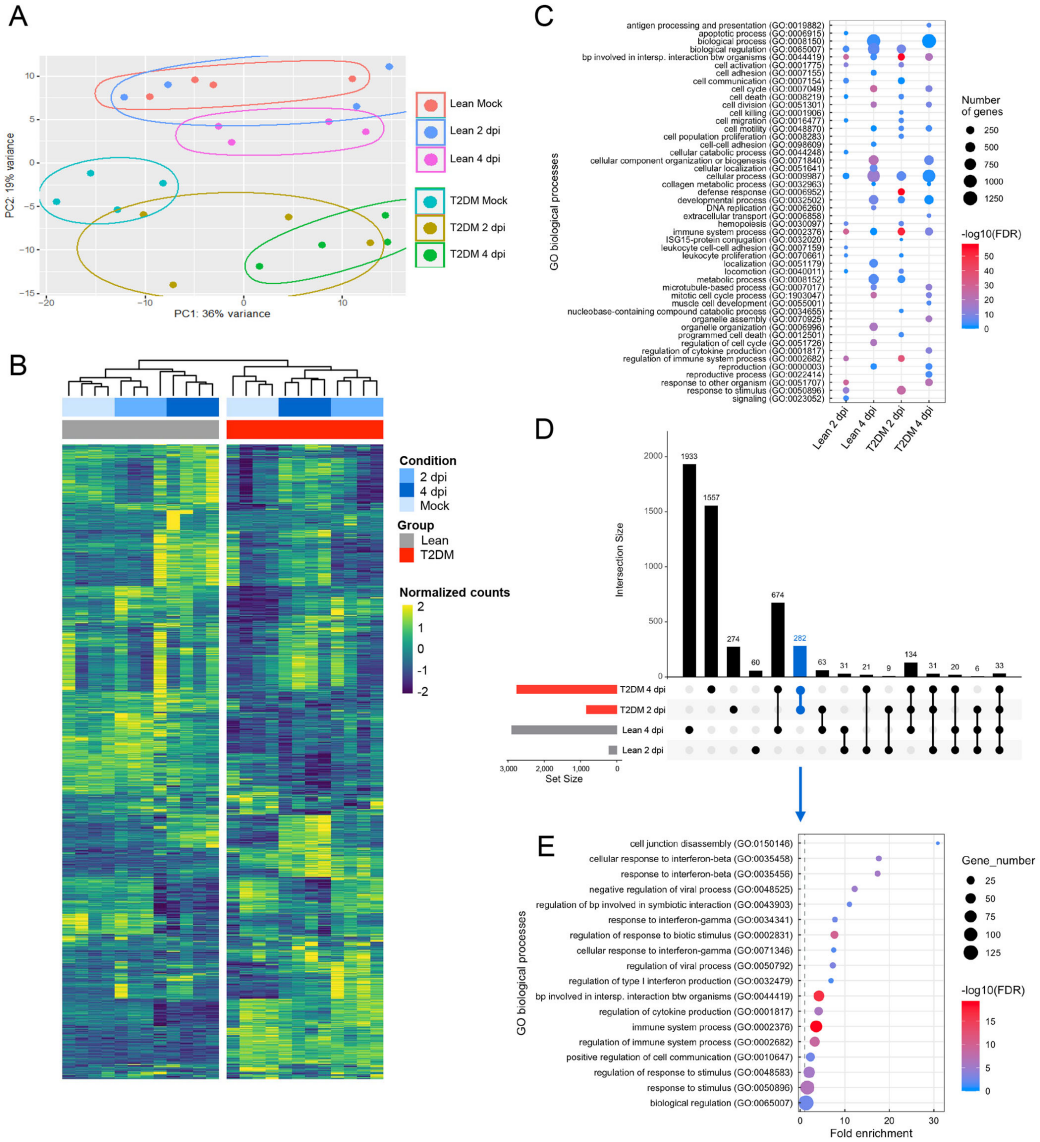
*Lepr*-deficient, T2DM mice have an exacerbated interferon beta and cytokine response following SARS-CoV-2 infection. **(A)** Principal component analysis (PCA) shows separate clusters between *Lepr*-deficient, T2DM mice, and lean counterparts. A high overlap was observed between mock-infected lean mice and lean mice infected at 2 dpi. Each dot indicates a single sample colored by group. **(B)** Heatmap showing the total differentially expressed genes (DEGs) present in the lungs of mock and SARS-CoV-2 infected *Lepr*-deficient, T2DM, and lean mice at 2 dpi and 4 dpi. Rows and columns were clustered using Euclidean distance. Samples were categorized automatically according to condition (Mock, 2 dpi, and 4 dpi) and group (Lean, T2DM). **(C)** Gene ontology (GO) biological processes significantly upregulated in the lungs of *Lepr*-deficient, T2DM mice at 2 dpi; *Lepr*-deficient, T2DM mice at 4 dpi; lean mice at 2 dpi and lean mice at 4 dpi. Each condition is compared to its respective mock-infected control. **(D)** UpSet plot summarizing the number of up-regulated DEGs in the lungs of lean and *Lepr*-deficient, T2DM mice at 2 dpi and 4 dpi when compared to their respective mock-infected control. The number of up-regulated DEGs in each group or their combination is shown as vertical bars. The blue vertical bar represents the number of DEGs up-regulated in the lungs of *Lepr*-deficient, T2DM mice at 2 dpi and 4 dpi but not in the lean mice at 2 dpi and 4 dpi. The bottom left horizontal bar graph labeled Set Size shows the total number of DEGs in the lungs of *Lepr*-deficient, T2DM mice at 2 dpi, *Lepr*-deficient, T2DM mice at 4 dpi, lean mice at 2 dpi and lean mice at 4 dpi. **(E)** GO biological processes significantly upregulated only in *Lepr*-deficient, T2DM mice at 2 dpi and 4 dpi. The up-regulated GO biological processes include those associated with the response to interferon-beta, the response to interferon-gamma, and immune system processes.

To elucidate differences in pulmonary responses between lean and *Lepr*-deficient, T2DM mice to SARS-CoV-2 infection, we performed global transcriptome analysis of the lung at 2 dpi and 4 dpi and compared them to the respective mock-infected animals. PCA revealed clear clustering between lean and *Lepr*-deficient, T2DM mice (**Figure 4A**). A high overlap was observed between mock-infected lean mice and lean mice infected at 2 dpi, which may be explained by the lack of detectable infectious virus and low levels of viral RNA at this time point in 3/4 (75%) of the infected lean mice (**Figure 4A**). Based on the hierarchical clustering of DEGs, the physiological status of these mice (i.e., lean vs *Lepr*-deficient, T2DM) had a significant impact on genes’ clustering followed by the infection status (i.e., mock, 2 dpi, and 4 dpi) (**Figure 4B**). GO biological process analysis revealed differences in overexpressed biological processes between lean- and *Lepr*-deficient, T2DM-infected mice at 2 dpi and 4 dpi (**Figure 4C**). A higher expression of genes mostly involved in immune system-related processes was observed at 2 dpi in infected *Lepr*-deficient, T2DM mice, as well as pathways associated with ISG15-protein conjugation and defense response. As we aimed to identify immune signatures correlated with exacerbated SARS-CoV-2 replication and delayed clearance in *Lepr*-deficient, T2DM mice, we then evaluated DEGs specifically expressed in each group and at specific time points after infection. Several DEGs were uniquely observed in the lung of lean mice at 4 dpi (1,933 DEGs) and *Lepr*-deficient, T2DM mice at 4 dpi (1,557 DEGs), while only 274 DEGs and 60 DEGs were uniquely observed for *Lepr*-deficient, T2DM mice at 2 dpi and lean mice at 2 dpi, respectively (**Figure 4D**). GO biological process analysis was subsequently performed when taking into account the 282 DEGs uniquely identified in *Lepr*-deficient, T2DM mice at 2 and 4 dpi and results demonstrated an increase of biological processes related to interferon beta (IFNβ) responses, IFNγ, regulation of viral process and cytokine production (**Figure 4E**). These data are correlated with Ingenuity Pathway Analysis (IPA), where eleven canonical pathways were upregulated in the lung of *Lepr*-deficient, T2DM mice compared to lean mice at 2 and 4 dpi (**Supplementary Figure S6A**), including “pathogen induced cytokine storm signaling pathway” characterized by an overexpression of *Zbp1*, *Ccl2*, *Irf7*, *Cxcll9*, *Cxcl10* and *Il6* (**Supplementary Figure S6B**), the “role of hypercytokinemia/hyperchemokinemia in the pathogenesis of Influenza” pathway characterized by an overexpression of *Irf7*, *Isg15*, *Oas3*, *Oas2*, *Rsad2*, *Ccl2* and *Il6* (**Supplementary Figure S6C**) and the “interferon signaling” pathway characterized by overexpression of *Isg15*, *Socs1*, *Stat1*, *Tap1*, *Stat2* and *Irf9* (**Supplementary Figure S6D**).

**Figure 5:**
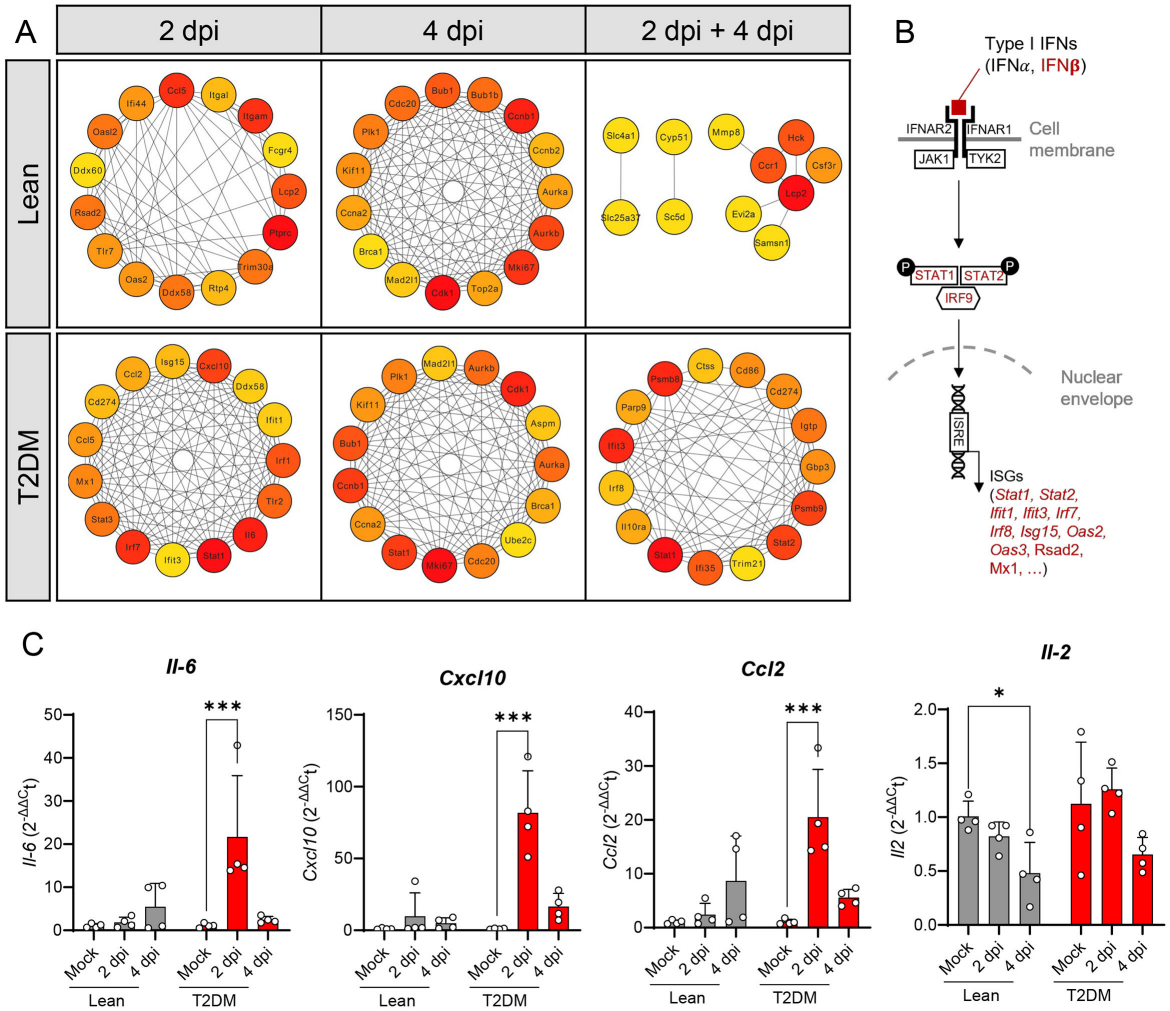
“Hub” gene identification by STRING analysis showing activation of type I interferon signaling pathway and cytokine storm in the lung of SARS-CoV-2 infected *Lepr*-deficient, T2DM mice. **(A)** Identification of the top 15 “hub” genes among upregulated genes using Cytohubba plugin in Cytoscape software. The image shows the degree of importance of hubs through a color scale ranging from red (the most important) to yellow (less important). **(B)** Schematic representation of the type I interferon pathways. The genes in red are significantly upregulated in the lungs of T2DM mice after SARS-CoV-2 infection. **(C)** Relative gene expression of four pro-inflammatory chemokines in the lungs of lean and *Lepr*-deficient, T2DM mice at 2 dpi and 4 dpi. *Il-6*, *Cxcl10*, and *Ccl2* are up-regulated in the lung of *Lepr*-deficient, T2DM mice at 2 dpi, while no change was observed for *Il-2.* Bars represent the mean ± standard deviation. *P ≤ 0.05; ***P ≤ 0.001.

STRING analysis was performed to identify the “Hub gene” sets upregulated in both groups of mice at 2 dpi and 4 dpi. “Hub gene” sets identified in the lean mice at 2 dpi and 4 dpi are different from those identified in *Lepr*-deficient, T2DM mice (**Figure 5A**). Although there are few genes related to the type I IFN response and the protection against viral RNA infection in the lean mice (e.g., *Oas2*, *Ifit44* and *Oasl2*), those were only observed at 2 dpi and were not present at 4 dpi. However, several genes involved in the type I interferon signaling pathway (*Isg15, Stat1, Stat2, Ifit3, Irf7*) and in the cytokine storm pathway (*Il6, Ccl2, Ccl6* and *Cxcl10*) were present in the “Hub gene” sets of *Lepr*-deficient, T2DM mice at 2 dpi and 4 dpi (**Figures 5A, B**; **Supplementary Figure S7**). Overexpression of *Il6, Ccl2* and *Cxcl10*, but not *Il2*, was confirmed by qPCR in the lung of *Lepr*-deficient, T2DM mice (**Figure 5C**).

**Figure 6:**
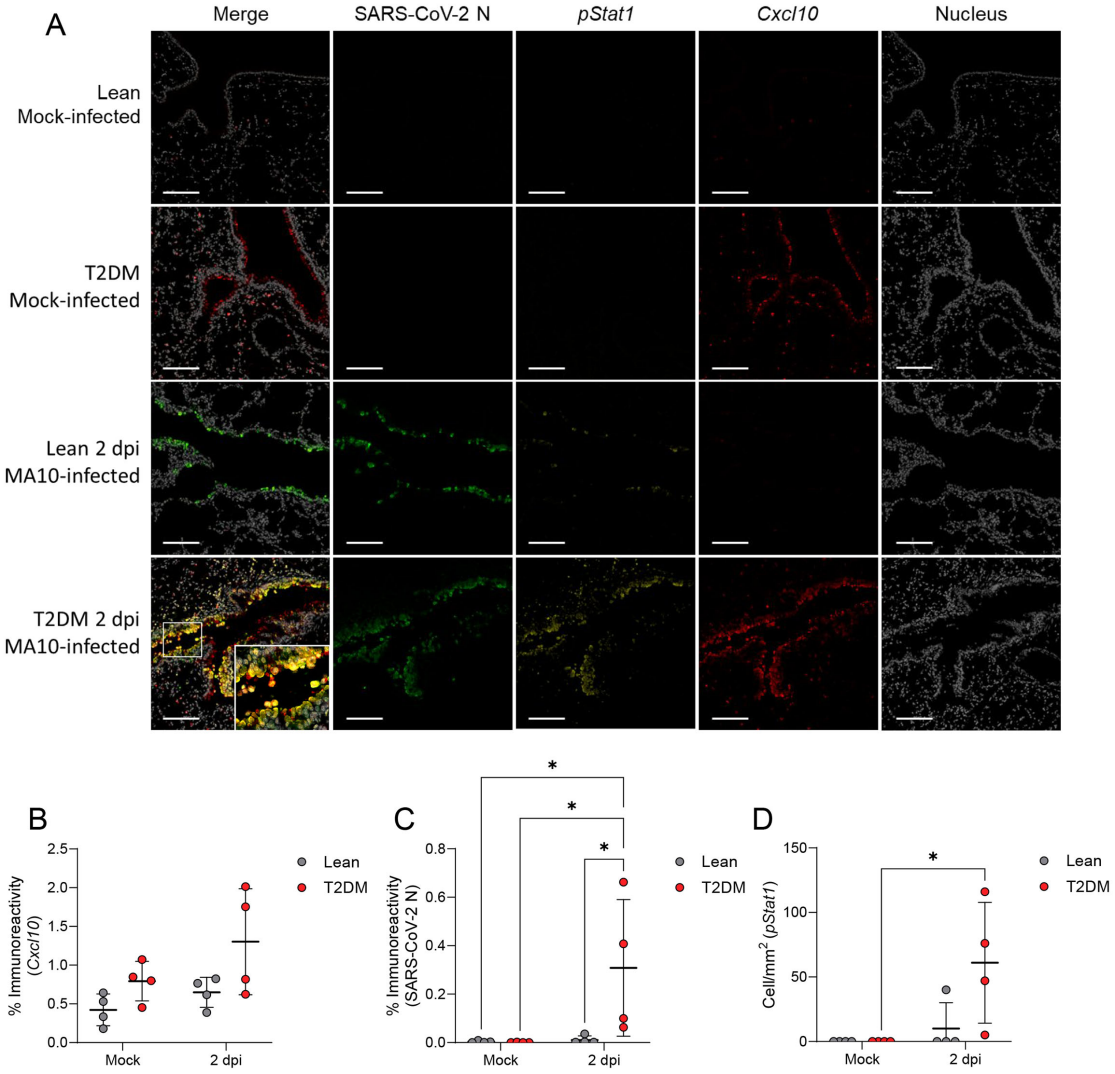
*Lepr*-deficient, T2DM mice show increased STAT1 phosphorylation (pSTAT1) and CXCL10 in airway epithelia following SARS-CoV-2 infection. **(A)** Representative multiplex immunofluorescence staining of Mock-infected, and SARS-CoV-2-infected lean and *Lepr*-deficient, T2DM mice at 2 dpi (Scale bar: 100 μm). Quantification of CXCL10 **(B)**, SARS-CoV-2 N **(C)**, and phosphorylated STAT1 (pSTAT1) **(D)** expression in the lungs of lean and *Lepr*-deficient, T2DM mice demonstrated pSTAT1 activation in response to SARS-CoV-2 infection in the lungs of *Lepr*-deficient, T2DM mice, but not in lean mice. pSTAT1 and CXCL10 in *Lepr*-deficient, T2DM mice are strongly expressed by bronchiolar epithelial cells (Scale bar: 100 μm). Bars represent the mean ± standard deviation. *P ≤ 0.05.

**Figure 7:**
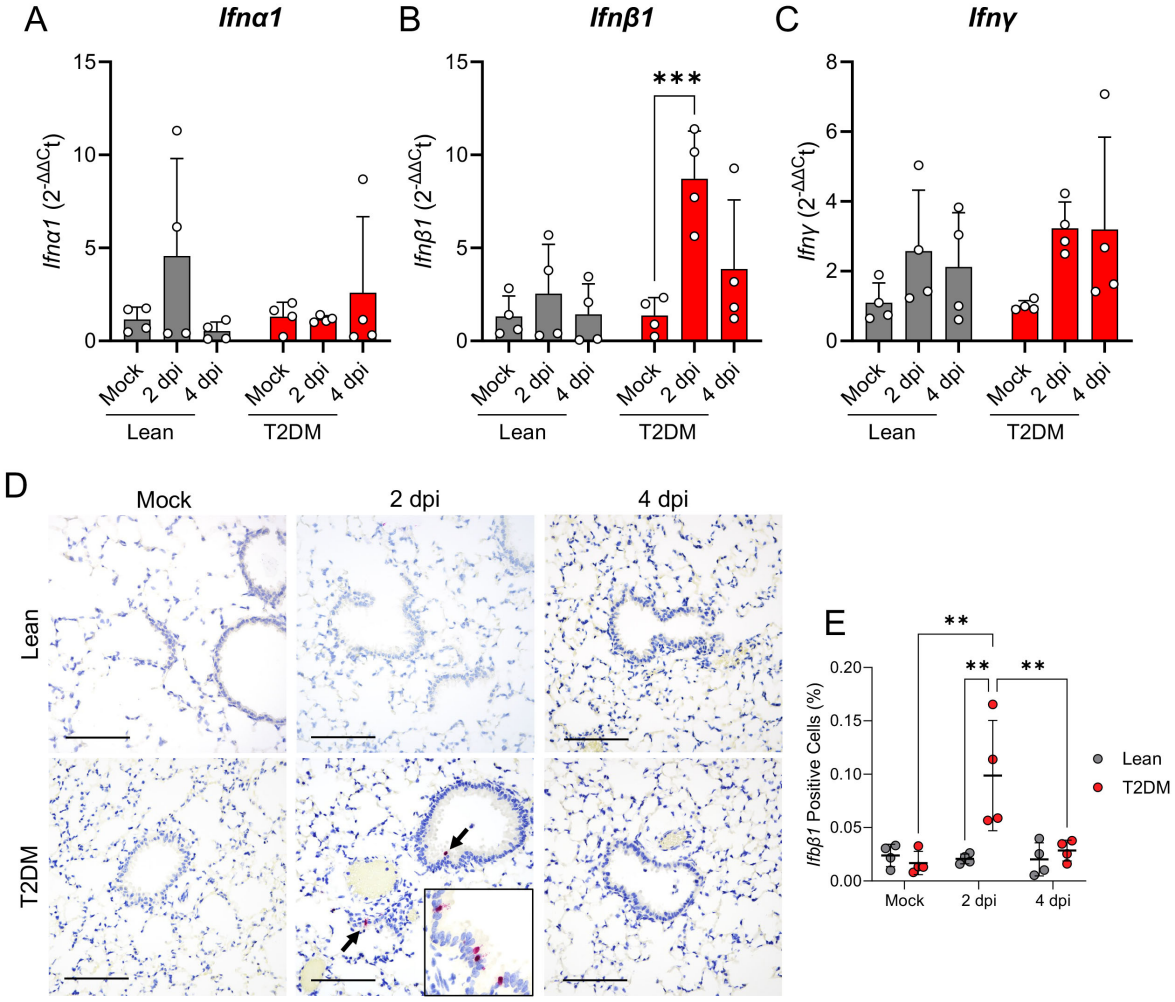
*Lepr*-deficient, T2DM mice show an exacerbated IFN beta pulmonary response following SARS-CoV-2 infection. Relative gene expression of *Ifna1* **(A)**, *Ifnb1* **(B)**, and *Ifng* **(C)** in the lungs of mock-infected and SARS-CoV-2-infected lean and *Lepr*-deficient, T2DM mice at 2 dpi and 4 dpi revealed the activation of *Ifnb1*, but not *Ifna1* and *Ifng*. **(D)** Distribution and **(E)** abundance of *Ifnb1* mRNA in the lung of *Lepr*-deficient, T2DM, and lean mice by RNAscope^®^ ISH. *Ifnb1* mRNA is primarily expressed in the cytoplasm and nucleus of bronchiolar epithelial cells. × 200 total magnification (Scale bar: 200 μm). Bars represent the mean ± standard deviation. **P ≤ 0.01; ***P ≤ 0.001.

To correlate these transcriptomic differences to the spatial context of these pulmonary responses, we employed multiplex fluorescent immunohistochemistry (mfIHC) targeting SARS-CoV-2 nucleocapsid (N), CXCL10, phosphorylated STAT1 (pSTAT1; Tyr701), pSTAT3 (Tyr705) and pSTAT5 (Tyr694), in addition to RNAscope^®^ *in situ* hybridization targeting *Ifnb1* mRNA in the lungs of infected mice. Interestingly, CXCL10 expression was higher in mock-infected *Lepr*-deficient, T2DM mice compared to lean counterparts, and its expression increased by 2 dpi primarily in bronchiolar epithelial cells and scattered infiltrating mononuclear cells (**Figures 6A, B**). pSTAT1 was significantly elevated in *Lepr*-deficient, T2DM mice (pSTAT1; *P* ≤ 0.001), and co-localized with SARS-CoV-2-infected bronchiolar epithelial cells (**Figures 6A, C, D**). In contrast, pSTAT1 expression in lean mice was negligible. Since STAT1 is activated downstream of type I (IFNα, IFNβ) and type II (IFNγ) interferon pathways, we analyzed the gene expression of *Ifna1*, *Ifnb1*, and *Ifng* by qPCR (**Figures 7A–C**). Only *Ifnb1* was significantly and specifically up-regulated in the lungs of *Lepr*-deficient, T2DM mice at 2 dpi (*P* ≤ 0.001). We determined that expression of *Ifnb1* by RNAscope^®^ *in situ* hybridization in the lung of SARS-CoV-2-infected *Lepr*-deficient, T2DM mice involved bronchiolar epithelial cells, with both intranuclear and intracytoplasmic signal detection (*P =* 0.001; **Figures 7D, E**). We analyzed *pStat3* and *pStat5a/b* gene and protein expression, as they are part of the *Lepr* signaling pathway and type I interferon response. While RNAseq showed slight *Stat3* overexpression in *Lepr*-deficient, T2DM mice (**Supplementary Figures S8A–S8C**), there was no significant increase in pSTAT3 or pSTAT5 protein levels (**Supplementary Figure S8D**). Leptin, however, was highly overexpressed in the lungs of SARS-CoV-2-infected *Lepr*-deficient, T2DM mice at 4 dpi (P ≤ 0.001; **Supplementary Figure S5C**).

In conclusion, SARS-CoV-2 induces a unique and exacerbated pulmonary cytokine response and STAT1 activation in *Lepr*-deficient, T2DM mice, without activating STAT3 or STAT5. Overall, this response is ineffective in controlling early viral replication in the lungs of *Lepr*-deficient, T2DM mice.

### 3.5 SARS-CoV-2 induces a delayed N-specific CD4+ T lymphocyte and a prolonged S-specific CD8+ T lymphocyte response in *Lepr*-deficient, T2DM mice

**Figure 8:**
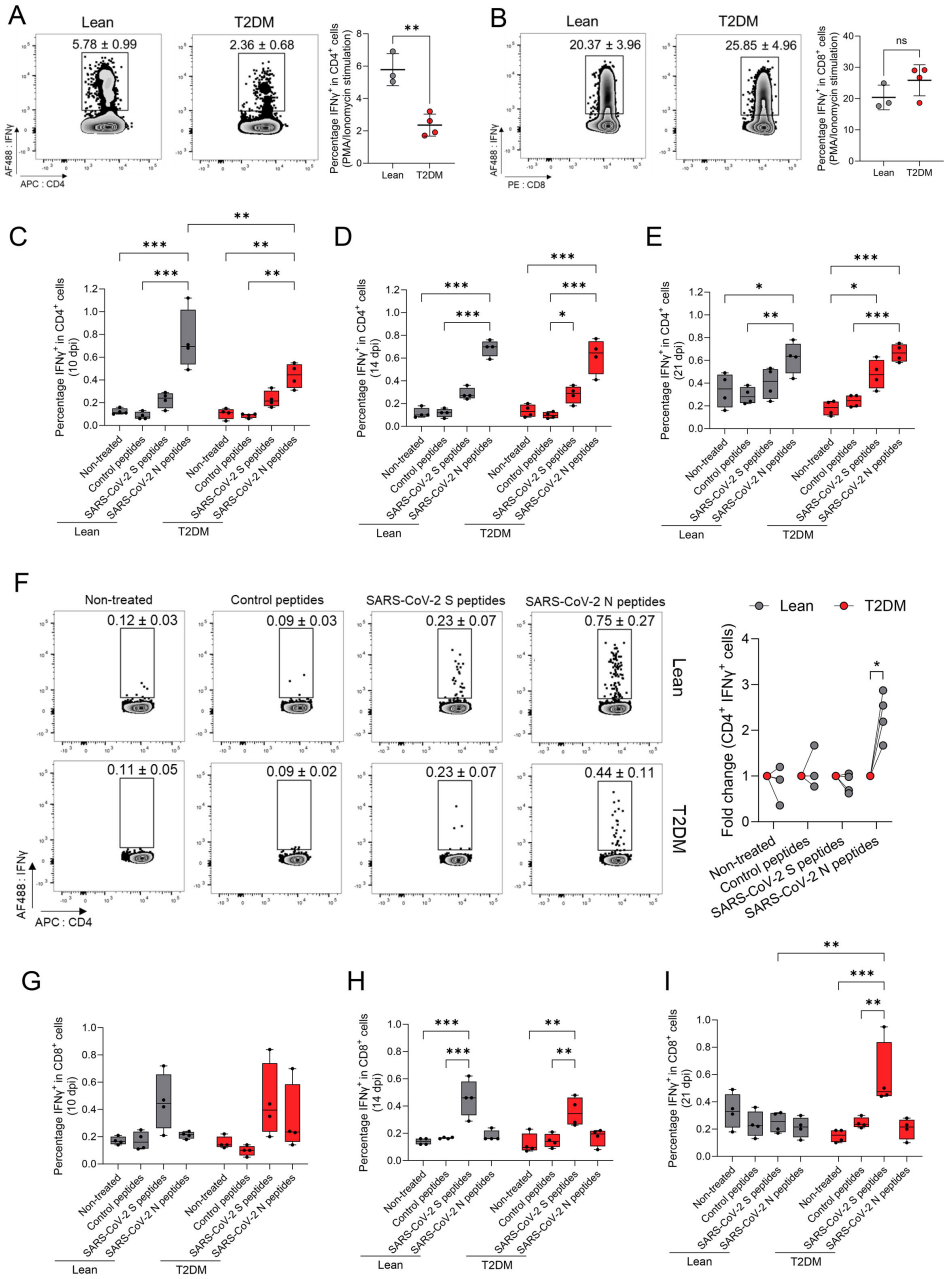
SARS-CoV-2-specific cell-mediated immune responses are delayed in the *Lepr*-deficient, T2DM mice. Percentage of splenic CD4^+^ **(A)** and CD8^+^ **(B)** lymphocytes T cells, collected from mock-infected lean and *Lepr*-deficient, T2DM mice producing IFNg after 5h of stimulation with a mix of PMA/ionomycin. A lower number of splenic CD4^+^ IFNg^+^ T lymphocytes is observed in *Lepr*-deficient, T2DM mice when compared to lean mince. Bars represent the mean ± standard deviation. Percentage of splenic CD4^+^ lymphocyte T cells collected at 10 dpi **(C)**, 14 dpi **(D)**, and 21 dpi **(E)** and producing IFNg after 5h of stimulation with SARS-CoV-2 spike (S), nucleocapsid (N) or control peptides. **(F)** Representative FACS plots of gated splenic CD4^+^ lymphocytes T cells from lean mice (upper graphs) and *Lepr*-deficient, T2DM mice (lower graphs) producing IFNg at 10 dpi. Percentage of splenic CD8^+^ lymphocytes T cells collected at 10 dpi **(G)**, 14 dpi **(H)**, and 21 dpi **(I)** and producing IFNg after 5h of stimulation with SARS-CoV-2 spike (S), nucleocapsid (N) or control peptides. Box plots represent the median value with the upper and lower quartile values, and the extended lines (whiskers) indicate the upper and lower extreme values. *P ≤ 0.05; **P ≤ 0.01; ***P ≤ 0.001; ns: non-significant difference (*P* > 0.05).

Next, we investigated whether cell-mediated immune (CMI) responses to SARS-CoV-2 differed between *Lepr*-deficient, T2DM and lean mice, by analyzing murine splenic T-lymphocyte (CD4^+^ and CD8^+^)-specific responses. First, to determine whether IFNγ production by T cells differed between *Lepr*-deficient, T2DM and lean mice at baseline, we stimulated splenocytes from mock-infected *Lepr*-deficient, T2DM and lean mice with a mixture of Phorbol 12-Myristate 13-Acetate (PMA) and ionomycin for 5 hours (h), and subsequently analyzed IFNγ-producing CD8^+^ and CD4^+^ T lymphocytes by flow cytometry. We determined a significantly lower percentage of CD4^+^ IFNγ^+^ T lymphocytes in *Lepr*-deficient, T2DM mice compared to their lean counterparts (*P* = 0.003; **Figure 8A**). No significant differences in the percentage of CD8^+^ IFNγ^+^ T lymphocytes were noted (*P* = 0.178; **Figure 8B**). These findings suggest differences in CD4+ T lymphocyte stimulation between *Lepr*-deficient, T2DM and lean mice at baseline.

Next, splenocytes collected from both groups of mice at 10, 14 and 21 dpi were stimulated for 5h with SARS-CoV-2-specific spike (S) glycoprotein and nucleocapsid peptides to characterize SARS-CoV-2-specific CMI responses. The percentage of both CD4^+^ IFNγ^+^ and CD8^+^ IFNγ^+^ T lymphocytes post-stimulation was determined by flow cytometry. Interestingly, when assessing SARS-CoV-2-specific CD4^+^ T cell responses, no spike-specific CD4^+^ T lymphocyte responses were identified throughout the course of the experiment in either *Lepr*-deficient, T2DM nor lean mice (**Figures 8C–E**). Conversely, when compared with the control peptides, we observed a significant increase in the percentage of CD4^+^ IFNγ^+^ in response to SARS-CoV-2 N-specific peptides starting at 10 dpi for both lean (*P* ≤ 0.001) and *Lepr*-deficient, T2DM (*P* = 0.003) mice (**Figure 8C**). A significantly higher percentage of SARS-CoV-2-N-specific CD4^+^ IFNγ^+^ T lymphocytes (fold change [1.83 ± 0.086]) were identified at 10 dpi in lean mice when compared to *Lepr*-deficient, T2DM mice (*P* = 0.01; **Figure 8F**). Between 14 and 21 dpi, no statistically significant differences in the percentage of SARS-CoV-2-N-specific CD4^+^ IFNγ^+^ T lymphocytes were evident between the two groups. These findings indicate a delayed SARS-CoV-2-specific CD4^+^ T lymphocyte response in the *Lepr*-deficient, T2DM mice compared to lean counterparts. In contrast, we did not detect a specific CD8^+^ T lymphocyte response following stimulation with SARS-CoV-2 N peptides (**Figures 8G–I**). Although a comparable SARS-CoV-2-S-specific CD8+ T lymphocyte response was detected at 14 dpi in both groups of mice (P ≤ 0.001; **Figure 8H**), it was short-lived in the lean mice compared to the *Lepr*-deficient, T2DM mice, in which it persisted until 21 dpi (*P* = 0.007; **Figure 8I**). In summary, SARS-CoV-2 triggered N-specific CD4+ and S-specific CD8+ T lymphocyte responses in both *Lepr*-deficient, T2DM and lean mice. However, these responses exhibited distinct characteristics, with the former being notably delayed and the latter lasting longer in *Lepr*-deficient, T2DM mice compared to their lean counterparts.

### 3.6 SARS-CoV-2 induces differential virus-specific antibody isotype responses in *Lepr*-deficient, T2DM mice

**Figure 9:**
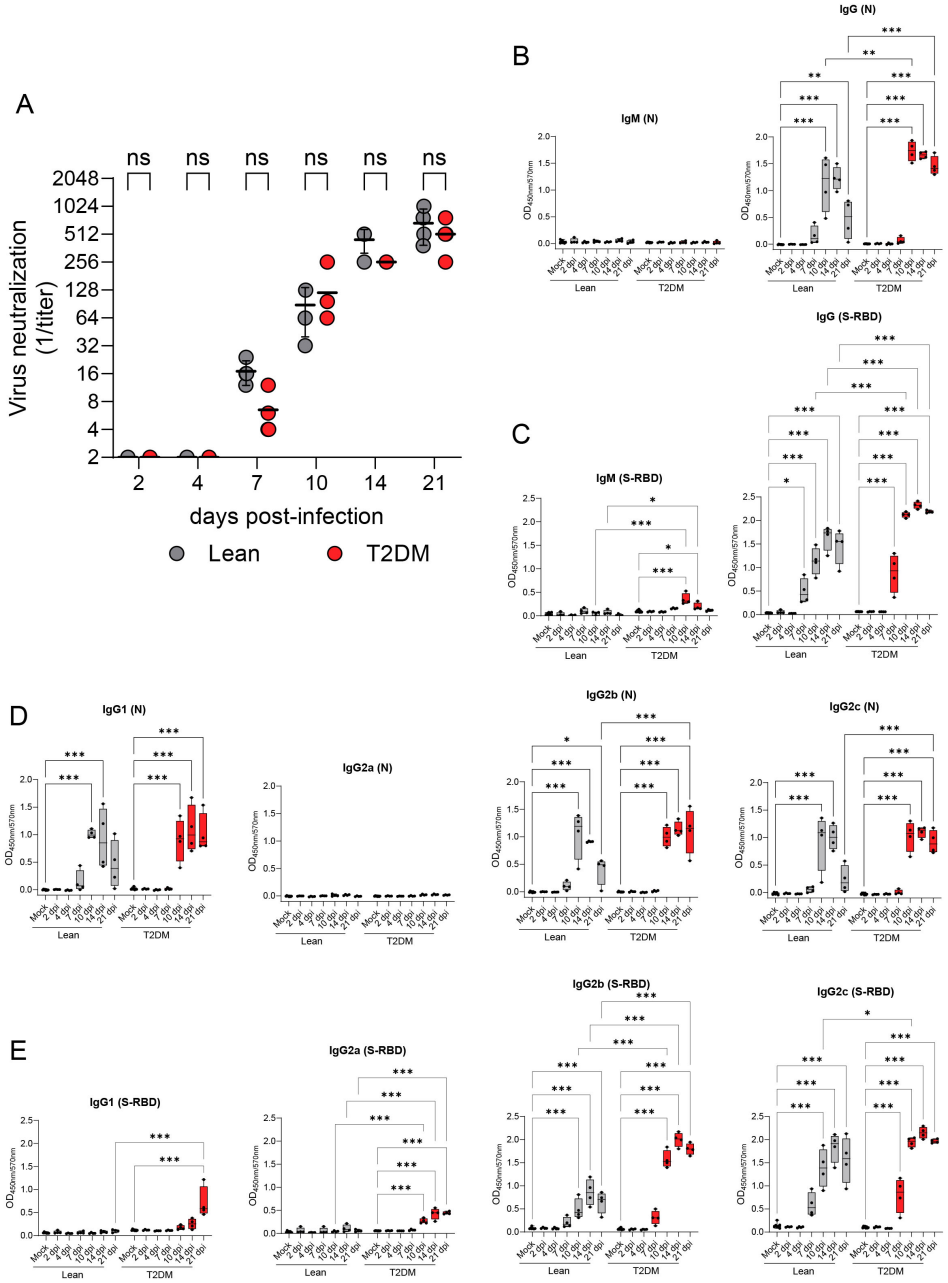
Differential serological immune responses to SARS-CoV-2 infection in *Lepr*-deficient, T2DM mice compared to lean mice. **(A)** Neutralizing antibody titers against SARS-CoV-2 MA10 strain measured in the serum of lean and *Lepr*-deficient, T2DM mice from 2 dpi to 21 dpi. Neutralizing antibodies were detected from 7 days onwards and increased likewise in both groups of mice. Horizontal bars represent the geometric mean ± geometric standard deviation. No significant differences (ns) were observed between lean and *Lepr*-deficient, T2DM mice for all time points. **(B)** SARS-CoV-2 nucleocapsid (N)-specific IgM and IgG titer measured in the serum of lean and *Lepr*-deficient, T2DM mice by indirect ELISA. **(C)** SARS-CoV-2 receptor binding domain (RBD)-specific IgM and IgG titers measured in the serum of lean and *Lepr*-deficient, T2DM mice by indirect ELISA. **(D)** SARS-CoV-2 N-specific IgG subtypes (IgG1, IgG2a, IgG2b, and IgG2c) titers measured in the serum of lean and *Lepr*-deficient, T2DM mice by indirect ELISA. **(E)** SARS-CoV-2 RBD-specific IgG subtypes (IgG1, IgG2a, IgG2b, and IgG2c) titers were measured in the serum of lean and *Lepr*-deficient, T2DM mice by indirect ELISA. Absorbance was read at 450 nm and 570 nm (OD_450nm/570nm_). Box plots represent the median value with the upper and lower quartile values, and the extended lines (whiskers) indicate the upper and lower extreme values. *P ≤ 0.05; **P ≤ 0.01; ***P ≤ 0.001; ns: non-significant difference (*P* > 0.05).

We first evaluated differences in neutralizing antibody levels elicited in *Lepr*-deficient, T2DM mice compared to lean counterparts. Neutralizing antibodies against SARS-CoV-2 were detected as early as 7 dpi in both groups of mice (**Figure 9A**) and neutralizing titers increased throughout the study, without significant differences between both groups. Subsequently, we characterized the serum immunoglobulin M (IgM) and IgG profiles elicited against SARS-CoV-2 N and SARS-CoV-2 S-RBD by indirect ELISAs (**Figures 9B, C**). While a SARS-CoV-2 N-specific IgM response was undetectable in both *Lepr*-deficient, T2DM, and lean mice (**Figure 9B**), there was a transient (short-lived) SARS-CoV-2 RBD-specific IgM response at 10 dpi (*P* ≤ 0.001) and 14 dpi (*P* = 0.05) only identified in *Lepr*-deficient, T2DM mice (**Figure 9C**). In both groups of mice, SARS-CoV-2 N-specific IgG and SARS-CoV-2 S-RBD-specific IgG responses were initially detected at 10 dpi and 7 dpi, respectively (**Figures 9B, C**). Notably, *Lepr*-deficient, T2DM mice exhibited significantly higher levels of SARS-CoV-2 N- and S-RBD-specific IgG compared to their lean counterparts. Specifically, elevated levels of SARS-CoV-2 N-specific IgG were detected in these mice at 10 dpi (P = 0.002) and 21 dpi (P ≤ 0.001), while higher levels of SARS-CoV-2 S-RBD-specific IgG were observed at 10 dpi (P ≤ 0.001), 14 dpi (P ≤ 0.001), and 21 dpi (P ≤ 0.001). In contrast, in lean mice, SARS-CoV-2 N-specific IgG levels significantly decreased by 21 dpi (P ≤ 0.001; **Figure 9B**), unlike the persistent dynamics seen in the *Lepr*-deficient group. We further characterized IgG subclass (IgG1, IgG2a, IgG2b, and IgG2c) dynamics following SARS-CoV-2 infection (**Figures 9D, E**). Among the IgG subclasses elicited against SARS-CoV-2 N, both groups of mice similarly elicited an N-specific IgG1, IgG2b and IgG2c response that correlated with the total N-specific IgG response (with a specific reduction of IgG subclass levels in lean mice compared to *Lepr*-deficient, T2DM mice at 21 dpi), while no N-specific IgG2a subclass response was induced (**Figure 9D**). The IgG subclass response specific to S-RBD was primarily mediated by IgG2b and IgG2c subclasses in both groups, and their dynamics correlated with that of total S-RBD-specific IgG (**Figure 9E**). Interestingly, we detected S-RBD-specific IgG1 and IgG2a subclass responses only in *Lepr*-deficient, T2DM mice at 21 dpi (*P* ≤ 0.001) and starting at 10 dpi (*P* ≤ 0.001), respectively (**Figure 9E**).

### 3.7 Silencing of the leptin receptor enhances SARS-CoV-2 replication in human alveolar epithelial cells under high leptin concentration levels.

**Figure 10:**
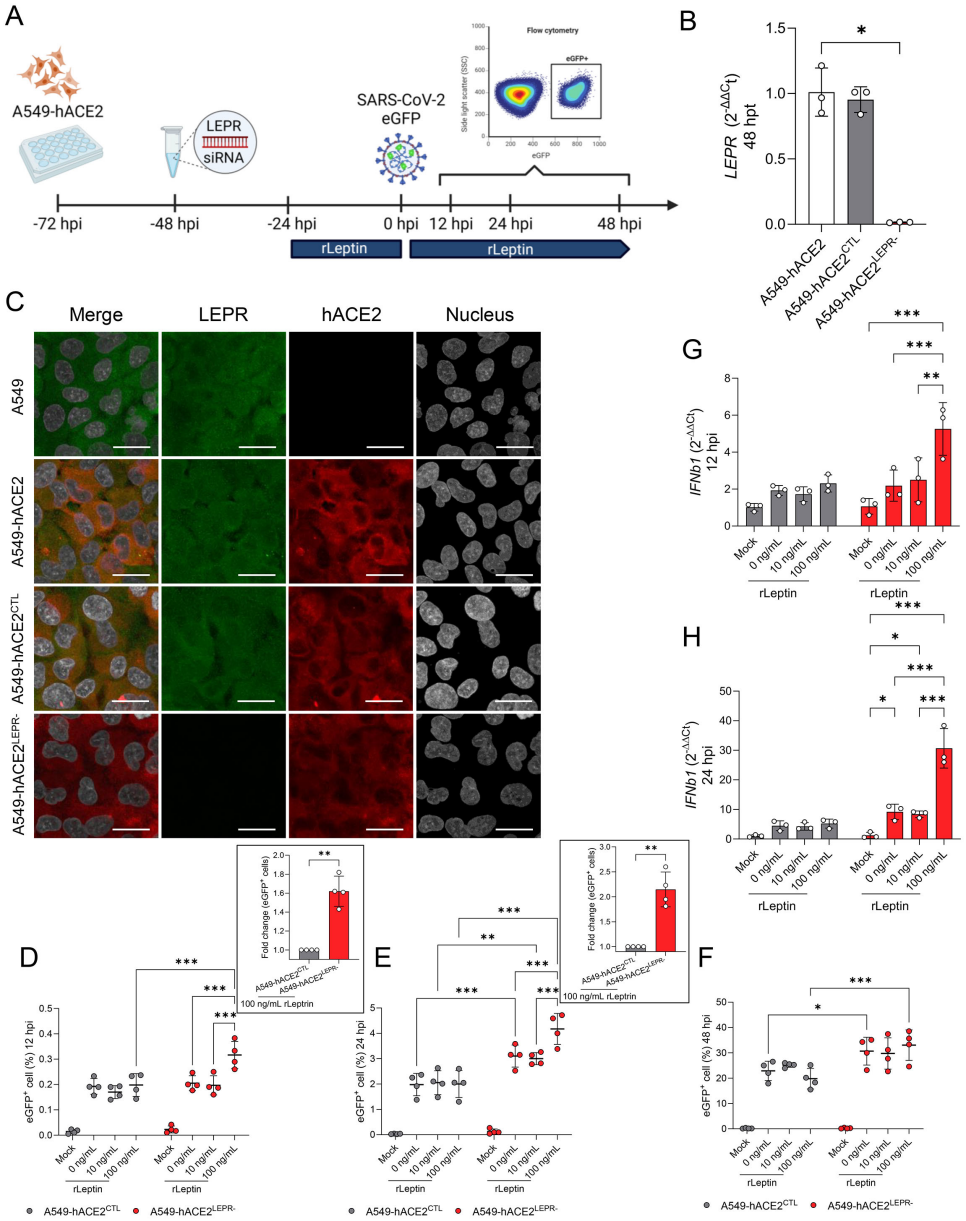
Silencing of the leptin receptor in A459-hACE2 cells enhances SARS-CoV-2 replication in the presence of high leptin levels. **(A)** Experiment design for evaluating the effect of LEPR-deficiency and high concentration of leptin on SARS-CoV-2 replication *in vitro*. A549-hACE2 were seeded on 24-well plates and treated with anti-LEPR siRNA (A549-hACE2^LEPR-^) or a control siRNA (A549-hACE2^CTL^), followed by the addition of recombinant leptin (rLeptin). Cells were infected with a recombinant SARS-CoV-2 USA-WA1/2020 expressing enhanced GFP (ic-SARS-CoV-2-eGFP) following the addition of recombinant leptin. Cells were harvested and the percentage of eGFP^+^ cells were counted by flow cytometry. **(B)** Relative gene expression of LEPR in A549-hACE2, A549-hACE2^LEPR-^ and A549-hACE2^CTL^ cells at 48h post-transfection. Bars represent the mean ± standard deviation. **(C)** Representative multiplex immunofluorescence staining of LEPR (green), human ACE2 (red), and nucleus (grey) in A549, A549-hACE2, A549-hACE2^LEPR-^ and A549-hACE2^CTL^ cells at 48h post-transfection (Scale bar: 20 μm). Percentage of eGFP^+^ A549-hACE2^LEPR-^ and A549-hACE2^CTL^ cells at 12 hpi **(D)**, 24 hpi **(E)**, and 48 hpi **(F)** in the presence of 0, 10, and 100 ng/ml of rLeptin. The insets illustrate the fold change of eGFP^+^ cells between A549-hACE2^LEPR-^ and A549-hACE2^CTL^, both treated with 100 ng/mL of rLeptin, at 12 and 24 hpi. Bars represent the mean ± standard deviation of four independent experiments performed in triplicate. Each dot represents the average of the triplicate. Relative gene expression of *IFNb1* in A549-hACE2^LEPR-^ and A549-hACE2^CTL^ at 12 hpi **(G)** and 24 hpi **(H)** demonstrated an increase of *IFNb1* expression in A549-hACE2^LEPR-^ and with concurrent administration of rLeptin. Bars represent the mean ± standard deviation. *P ≤ 0.05; **P ≤ 0.01; ***P ≤ 0.001.

Our *in vivo* studies in *Lepr*-deficient, T2DM mice demonstrated enhanced SARS-CoV-2 replication in the bronchiolar epithelium when compared to the lean mice. As a consequence of *Lepr* deficiency, these mice also exhibit hyperleptinemia (Kim et al., 2016). Hence, we wanted to determine the effect of the impaired leptin receptor signaling in combination with high levels of leptin on viral replication. We utilized a human alveolar epithelial cell line (A549-hACE2) that expresses both hACE2 (rendering it susceptible to SARS-CoV-2) and LEPR (**Figures 10A–C**). LEPR expression was specifically downregulated using siRNA treatment for 48h, then the recombinant human leptin (rLep) was added at 10 ng/mL (normal condition), and 100 ng/mL (hyperleptinemia) (Considine et al., 1996; Niskanen et al., 1997) for 24 hours before infection (**Figure 10A**). Cells were infected with a recombinant clone of the SARS-CoV-2 USA-WA1/2020 strain expressing eGFP (MOI = 0.1) and the percentage of cells expressing eGFP was monitored at 12 hpi, 24 hpi, and 48 hpi by flow cytometry (**Figures 10A–F**). The transfection of the A549-hACE2 cells with a siRNA targeting the LEPR (A549-hACE2^LEPR-^) induced a significant reduction in *LEPR* transcripts (*P* = 0.071; **Figure 10B**) when compared to the siRNA control (A549-hACE2^CTL^). In addition, immunofluorescence microscopy revealed high ACE2 expression in both A549-hACE2^LEPR-^ and A549-hACE2^CTL^ cells, while LEPR was undetectable in A549-hACE2^LEPR-^ cells (**Figure 10C**).

Rapidly after SARS-CoV-2 infection (12hpi), a significantly higher number of eGFP^+^ cells (fold change of 1.62 ± 0.16 [*P* = 0.004]) were observed in the A549-hACE2^LEPR-^ cells treated with 100 ng/mL of rLep (*P* ≤ 0.001; **Figure 10D**; **Supplementary Figure S9A**), when compared to the reciprocal A549-hACE2^CTL^ cells. No difference in the number of eGFP^+^ cells was observed between A549-hACE2^CTL^ cells (untreated or treated with rLep). Concomitantly, an increase in *IFNB1* mRNA expression was only observed in A549-hACE2^LEPR-^ cells treated with 100 ng/mL of rLep (*P* ≤ 0.003; **Figure 10G**). At 24 hpi, regardless of the concentration of rLep, the percentages of eGFP^+^ cells are significantly higher in the infected A549-hACE2^LEPR-^ when compared to infected A549-hACE2^CTL^ (*P* ≤ 0.003; **Figure 10E**; **Supplementary Figure S9B**). A fold change of 2.15 ± 0.35 (*P* = 0.007) was observed between A549-hACE2^CTL^ and A549-hACE2^LEPR-^, both treated with 100 ng/mL of rLeptin. Interestingly, while 10 ng/mL of rLep in A549-hACE2^LEPR-^ did not affect virus replication (*P* = 0.954), a ten-fold higher concentration of rLep induced a significant increase in the percentage of eGFP^+^ cells (*P* ≤ 0.001) at 24 hpi. While no increase of *IFNB1* expression has been noted in the A549-hACE2^CTL^ cells at 24 hpi when compared to the mock-infected cells (**Figure 10H**), a significant increase of *IFNB1* expression in A549-hACE2^LEPR-^ cells was observed in the absence (0 ng/mL; *P* = 0.013) and in presence of 10 ng/mL (*P* = 0.027) and 100 ng/mL of rLep (*P* ≤ 0.001). The expression of *IFNB1* is three-fold higher in the presence of 100 ng/mL of rLep when compared to 0 ng/ml and 10 ng/mL of rLep (*P* ≤ 0.001). At 48 hpi, the differences in the proportion of eGFP^+^ cells between A549-hACE2^LEPR-^ and A549-hACE2^CTL^ were significant with both lack of rLep and high concentrations of rLep (0 ng/mL; *P* = 0.013 and 100 ng/mL: *P* ≤ 0.001; **Figure 10F**). No differences were observed in the presence of physiological levels of rLep (10 ng/mL; *P* = 0.130). These results further support the important role of leptin and its receptor (LEPR) on SARS-CoV-2 infection and replication both *in vivo* and *in vitro*, and its interplay with IFNβ responses following infection.

## 4 Discussion

While it has been shown in three studies that hyperglycemic obese mice are more susceptible to SARS-CoV-2 than lean mice (Zhang et al., 2021; Lee et al., 2022; Johnson et al., 2023), our study critically adds novel information to the current body of knowledge as it represents the first study to thoroughly compare side-to-side the susceptibility of lean, DIO, and T2DM models (pre-diabetic vs diabetic) to SARS-CoV-2. Most importantly, this study sheds light on alterations in the innate immune pathway in the lungs through a comprehensive transcriptomic analysis and a detailed characterization of the specific humoral and cell-mediated immune responses of T2DM mice to SARS-CoV-2 infection. Our findings reveal for the first time that IFNβ plays a crucial role in the innate immune response and is linked to the leptin pathway, paving the way for further research into the relationship between these metabolic signaling pathways and the cellular response to infection.

Our team has previously described the low susceptibility of the B6 control mice (C57BL/6J) to the SARS-CoV-2 MA10 strain (Thieulent et al., 2023). In the current study, we have shown that inducing DIO in this mouse model did not result in clinical disease, enhanced viral replication, or enhanced pulmonary pathology following SARS-CoV-2 infection. In contrast to our study, another study using B6 DIO mice infected with the SARS-CoV-2 MA10 strain reported increased morbidity and mortality (Johnson et al., 2023). Key differences include the use of older mice (18 vs. 14 weeks) and a longer exposure to a high-fat diet (15 vs. 8 weeks), leading to hyperglycemia, a feature absent in our mice. This suggests that hyperglycemia (a hallmark of T2DM) rather than only overweight is likely a primary factor contributing to disease enhancement following SARS-CoV-2 infection.Consistent with our findings, SARS-CoV-2-infected male K18-hACE2 mice fed a high-fat diet for 8 weeks showed no increase in viral replication or clinical score (Lee et al., 2022). Moreover, previous studies have demonstrated that influenza A viruses (IAV) replicate at higher titers and induce more severe pulmonary lesions in various hyperglycemic mouse and ferret models (Reading et al., 1998; Tartof et al., 2020; Johnson et al., 2023; Meliopoulos et al., 2024). Elevated glucose levels before IAV infection also enhance virus-induced barrier damage and increase pro-inflammatory cytokines (Hulme et al., 2020).

To evaluate the effect of T2DM on SARS-CoV-2 infection, we used the BKS db/db mice, a well-characterized leptin receptor-deficient mouse model (Coleman, 1978; Chen et al., 1996). These mice developed subclinical SARS-CoV-2 MA10 infection but had increased lung viral replication and delayed clearance compared to lean mice, consistent with other glucose-intolerant obese models (Lee et al., 2022; Huang et al., 2023). However, despite the mouse model in this study not fully replicating the dynamics observed in humans, it is noteworthy that the delayed clearance in *Lepr*-deficient, T2DM mice aligns with findings in patients (Zhou et al., 2021; Zhang et al., 2022). The virus mainly targeted bronchiolar epithelium with limited alveolar infection, aligning with recent studies (Leist et al., 2020; Thieulent et al., 2023). To understand the increased viral replication and delayed clearance in *Lepr*-deficient, T2DM mice, we analyzed pulmonary host responses and adaptive cellular and humoral responses before and after infection. Firstly, the overall downregulation of genes linked to immune regulation, lymphocyte activation, and interferon activation and response at baseline in the lungs of non-infected *Lepr*-deficient, T2DM mice suggests a potential deficiency or impairment in lung immune responses. This is likely a key factor impeding early control of SARS-CoV-2 infection. Additionally, the lower number of splenic CD4^+^ T lymphocytes activated after PMA/ionomycin stimulation, along with a reduced count of SARS-CoV-2 N-specific splenic CD4^+^ T cells producing IFNγ at 10 dpi suggests a low-magnitude and/or impaired CD4^+^ T lymphocyte activation and function. Reduction of the function and increase of senescent T lymphocytes has been characterized in T2DM patients (Sheikh et al., 2018; Lau et al., 2019). Moreover, our observations are supported by another study, suggesting that leptin enhances CD4^+^ T lymphocyte differentiation and proliferation via leptin receptor signaling in a leptin-deficient mouse model, while no effect has been observed on CD8^+^ T lymphocyte proliferation (Kim et al., 2010).Using human monocytes, it was also demonstrated that high glucose levels directly enhance SARS-CoV-2 replication and increase proinflammatory cytokine expression. This occurs through a mitochondrial ROS/hypoxia-inducible factor-1α-dependent pathway, leading to T-cell dysfunction and epithelial cell death (Codo et al., 2020). These results indicate a potential deficit or dysfunction in immune responses in individuals with T2DM, likely serving as a primary factor hampering the control of SARS-CoV-2 infection.

Our data have demonstrated that lean mice control SARS-CoV-2 infection rapidly (≤ 2 dpi), while a prolonged infection has been observed in the lung of *Lepr*-deficient, T2DM mice (up to 7 dpi). Type I and type III IFNs constitute the primary line of immune defense against viral mucosal infections (Stanifer et al., 2020). Surprisingly, rapidly after infection (2 dpi), *Lepr*-deficient, T2DM mice exhibit an increased expression of IFNβ, but not IFNα, with apparent activation of JAK and phosphorylation of STAT1 (pSTAT1) leading to the expression of interferon-stimulated genes (ISGs). The absence of STAT1 activation at 2 dpi in the lean mice can be explained by either an even earlier activation (e.g., 1 dpi) or the rapid control of SARS-CoV-2 infection by other key factors. Despite triggering this defense mechanism, *Lepr*-deficient, T2DM mice are unable to control early SARS-CoV-2 replication. Type I IFN can also be induced by activating the cGAS/STING pathways through the release of mitochondrial DNA due to mitochondrial damage, leading to harmful rather than protective effects (Domizio et al., 2022). During SARS-CoV-2 infection, STING activation occurs in damaged lung epithelial cells and macrophages and is linked to increased pathology. Although an early and rapid induction of type I IFNs can limit virus replication, a sustained increase in their levels during later phases of infection is associated with aberrant inflammation and poor clinical outcomes (Lee et al., 2020; Domizio et al., 2022). A similar observation was made in a mouse model of SARS-CoV-2 infection based on adeno-associated virus (AAV)–mediated expression of hACE2 (Israelow et al., 2020). The exacerbated expression of IFNβ in the lung of *Lepr*-deficient, T2DM mice can therefore be one factor leading to prolonged SARS-CoV-2 infection. These data demonstrate the complex interaction between SARS-CoV-2, type I IFNs, and the overall local immune response. Additionally, the impact of DIO on immune dysregulation was not further investigated in the current study, as it has already been thoroughly examined in previous research (Lee et al., 2022). Lee and colleagues demonstrated greater overlap in pulmonary transcriptional changes between lean and DIO mice compared to mock and SARS-CoV-2-infected mice, indicating that viral infection has a more pronounced effect on the pulmonary transcriptome than diet. Notably, only the pathway associated with T-cell receptor signaling was found to be altered (Lee et al., 2022).

Interestingly, no significant activation of either STAT3 or STAT5, two downstream transcription factors of the leptin receptor signaling pathway (Darnell, 1996; Liu et al., 2021), was observed in either group of mice. Even though a lack of pSTAT3 and pSTAT5 was expected in *Lepr*-deficient, T2DM mice since they lack functional *Lepr*, there was also no compensatory activation of these STATs by the signaling mechanism driving STAT1 phosphorylation in these mice. The lean mice are *Lepr* competent, however, the absence of STAT3 and STAT5 phosphorylation could be associated with the physiologically low levels of circulating leptin, time of tissue collection, or other undetermined factors.

While *Lepr*-deficient, T2DM mice develop similar virus-neutralizing antibody titers to their lean counterparts, a divergence in the SARS-CoV-2-specific immunoglobulin profile has been observed between both groups of mice. It was demonstrated that the intensity of the serological response to SARS-CoV-2 is positively correlated with the severity of the clinical symptoms (Legros et al., 2021; Korobova et al., 2022; Wang et al., 2022; Yan et al., 2022), which explains why higher and prolonged SARS-CoV-2 N-specific IgG (IgG1, IgG2b, and IgG2c) levels were observed in the serum of the *Lepr*-deficient, T2DM mice when compared to the lean mice.

Finally, further understanding of the relationship between metabolic pathways in peripheral tissues (e.g., lung) and their influence on host responses to viral infections is critical to improving our knowledge of factors driving increased susceptibility in obese and T2DM patients and identifying appropriate therapeutic strategies for this at-risk population. While obesity and T2DM are complex and frequently polygenic diseases, leptin is a key regulator, and leptin resistance is a feature of these two metabolic diseases. The LEPR has a wide cellular expression, including pulmonary epithelia and immune cells (Busso et al., 2002; Nair et al., 2008; Vernooy et al., 2009). Preliminary data obtained in our laboratory shows that primary human bronchial/tracheal epithelial cells cultivated at the air-liquid interface express LEPR, and we have identified LEPR expression in lymphocytes within bronchial-associated lymphoid tissue, alveolar macrophages, and bronchial/bronchiolar epithelium. To date, mechanistic understanding of the effects that leptin has on host responses to respiratory viral infections leading to increased viral replication and disease severity in people with obesity and T2DM is not fully understood. Our study demonstrates that silencing of the leptin receptor, coupled with high leptin levels, increases SARS-CoV-2 replication and *IFNB* expression *in vitro* in a surrogate human alveolar-type cell line, similar to what we observed *in vivo*. A recent study established that leptin mediates the activation of IRF3 in hypothalamic neurons, a transcription factor that drives the expression of type I IFN and interferon-stimulated genes [ISGs] (Heyward et al., 2024). The signaling transduction mechanism for IRF3 phosphorylation via leptin remains undetermined and the authors suggested the involvement of PI3K-Akt as a poorly understood arm of the leptin signaling pathway. *Irf3* was not upregulated in the lungs of SARS-CoV-2 infected mice in our study, which could suggest another mechanism for IFNβ induction. However, we did not assess activation/phosphorylation status to rule out its possible involvement. This study establishes a relationship opening new avenues to investigate the specific role of the leptin receptor pathway in shaping host responses to viral infection. Altogether, our data suggests that modulating the leptin receptor signaling could, hypothetically, help restore immune homeostasis in T2DM patients, which might mitigate some of the complications associated with COVID-19 in this patient population. It has been shown that amylin-induced alternative phosphorylation of STAT3 restores leptin signaling (Roth et al., 2008; Le Foll et al., 2015) and so does the suppression of SOCS3 (Pedroso et al., 2014). Hence, it is possible to pharmacologically or genetically modulate the leptin receptor signaling and restore local and immunological responses to circumvent the exacerbated viral replication in the respiratory tract, leading to the identification of potential therapeutics for intervention. Further experiments using air-liquid interface cultures of primary large and small airway cells or the use of organoids, as well as additional animal models will be necessary to gain more insight into the role of this pathway in shaping cellular responses to viral infections such as SARS-CoV-2 or influenza virus A.

This study has some limitations. One is the use of the SARS-CoV-2 MA10 strain, which, despite mutations enabling it to bind murine ACE2 (Dinnon et al., 2020; Leist et al., 2020), did not induce clinical signs at the infectious dose used. This dose, based on our previous studies and early literature on this strain (Leist et al., 2020; Thieulent et al., 2023), was chosen to ensure a sublethal model for assessing adaptive immune responses. A higher dose might enhance some phenotypic features but limit the assessment of adaptive immune responses. Additionally, our study focused on Wuhan-like mouse-adapted strain, so evaluating SARS-CoV-2 variants of concern (VOC) to which mice are naturally susceptible (i.e., Alpha, Delta) and more recent VOCs (i.e., Omicron) is crucial (Chen et al., 2022; Stolp et al., 2022). Another limitation is the exclusive use of male mice, necessitating further studies with female *Lepr*-deficient, T2DM mice to explore potential sex differences. This study did not investigate viral replication in adipose tissues, although it has been observed in human patients (Zickler et al., 2022). However, the significance of this tissue in viral transmission remains low. Finally, our study primarily aimed to investigate the broader immunological and virological impacts of T2DM in SARS-CoV-2 infection, with an emphasis on identifying phenotypic changes and key pathways potentially contributing to increased viral replication and disease severity. Consequently, the relationship between leptin resistance and SARS-CoV-2 pathogenesis requires further investigation. Direct modulation of leptin receptor signaling using alternative activators and antagonists represents an important and complementary avenue for future research.

To conclude, this study provides further insight into host responses during SARS-CoV-2 infection, and more specifically under the effects of obesity and T2DM as comorbidities. Here, we demonstrated that T2DM influences local pulmonary host responses to SARS-CoV-2 and the CD4^+^ T lymphocyte-mediated immune response, leading to an increase in SARS-CoV-2 replication and a delay in virus clearance. The increased viral replication is, at least in part, associated with impaired leptin receptor signaling both *in vitro* and *in vivo*, and increased but ineffective IFNB responses. Further mechanistic studies to unravel the role of this signaling pathway in respiratory viral infections are warranted.

## Data Availability

The datasets presented in this study can be found in online repositories. The names of the repository/repositories and accession number(s) can be found below: https://www.ncbi.nlm.nih.gov/geo/, GSE270802.
