# Supplementary material for: Diabetes exacerbates SARS-CoV-2 replication through ineffective pulmonary interferon responses, delayed cell-mediated immunity, and disruption of leptin signaling
Source: Front Cell Infect Microbiol. 2025 Mar 7;15:1513687. doi: 10.3389/fcimb.2025.1513687 (PMC11925909; doi:10.3389/fcimb.2025.1513687)
Supplement: Supplementary file 1 [file SupplementaryFile1.docx]

Supplementary Material

# Supplementary Material 1: Multiplex fluorescent immunohistochemistry (mfIHC) and quantitative image analysis.

***SARS-CoV-2 N, CXCL10 and pSTAT1 mfIHC.*** A Ventana Discovery Ultra (Roche, Basel, Switzerland) tissue autostainer was used for brightfield and multiplex fluorescent immunohistochemistry (fmIHC). In brief tyramide signaling amplification (TSA) was used in an iterative approach to covalently bind Opal fluorophores (Akoya Bioscience, Marlborough, MA) to tyrosine residues in tissue, with subsequent heat stripping of primary-secondary antibody complexes until all antibodies were developed. Lungs from infected (positive controls) and uninfected (negative controls) mice were used as controls for assay optimization. Antigen retrieval was conducted using a Tris-based buffer-CC1 (Roche). Specific primary antibody dilutions are shown in Supplementary Table 1. Antibodies for STAT1-P, and CXCL10 were of rabbit origin, and thus developed with a secondary goat anti-rabbit HRP-polymer antibody (Vector Laboratories, Newark, CA) for 20 min at 37 °C. Primary antibody for SARS-CoV-2 N protein was of mouse origin and developed with a secondary goat anti-mouse HRP-polymer antibody (Vector Laboratories) for 20 min at 37 °C. All Opal TSA-conjugated fluorophore reactions took place for 20 minutes. Fluorescent slides were counterstained with spectral DAPI (Akoya Biosciences) for 16 minutes before being mounted with ProLong gold antifade (ThermoFischer).

***pSTAT1, pSTAT3 and pSTAT5 mfIHC.*** Following antibody optimization by singleplex chromogenic IHC for each of the antibodies included in the panel, an Akoya Opal 4-plex protocol was established for mfIHC and performed using the BOND RX^m^ platform (Leica Biosystems). Briefly, 4-micron formalin-fixed and paraffin-embedded tissue sections were subjected to automated baking, deparaffinization, and antigen retrieval using an EDTA-based pH 9.0 retrieval buffer at 100 °C for 20 min. Subsequently, sections were subjected to three sequential blocking, primary antibody incubation, secondary antibody incubation, Opal fluorochrome incubation and heat stripping. The blocking step was performed using Akoya’s blocking buffer (10 min, room temperature [RT]). Primary antibodies were diluted in Akoya’s antibody diluent and incubated for 30 min at RT. Specific primary antibody dilutions are shown in Supplementary Table 1. The secondary antibody step included a ready-to-use polymer-labeled goat anti-rabbit IgG conjugated with horseradish peroxidase (HRP) for 8 min at RT. Opal fluorophores were diluted following manufacturer's recommendation and incubated for 10 min at RT. Stripping was performed with a citrate-based pH 6.0 retrieval buffer for 20 min at 97 °C. After the final stripping step, tissue sections were incubated with Akoya’s spectral DAPI as instructed by the manufacturer for 5 min at RT. Slides were finally coverslipped with ProLong Diamond Anti-fade mounting solution (ThermoFisher).

Supplementary Figures and Tables

## Supplementary Figures


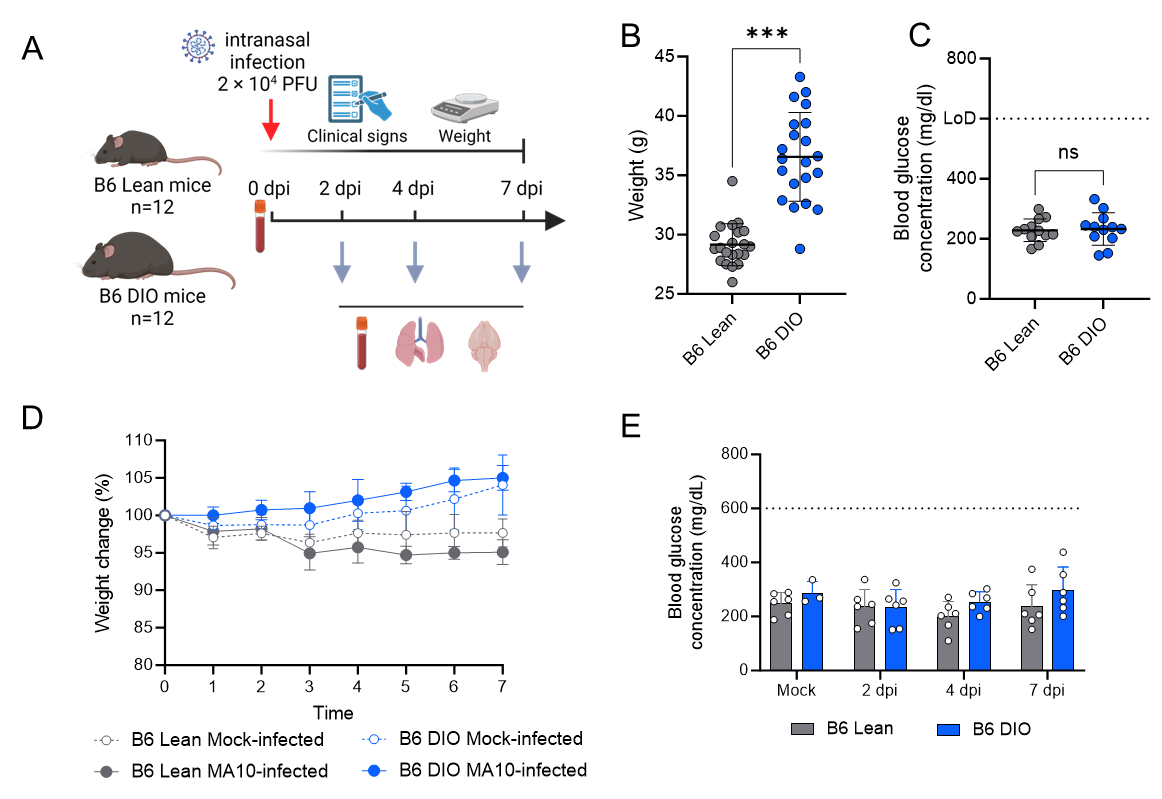


**Supplementary Figure 1.** **Diet-induced obesity under normoglycemic conditions does not enhance disease severity following SARS-CoV-2 MA10 infection.** (**A**) Experimental approach for evaluating the effect of diet-induced obesity (DIO) on SARS-CoV-2 infection. B6 DIO (n=9) and B6 lean (n=9) mice were intranasally infected with 2 × 10^4^ PFU of SARS-CoV-2 MA10 strain, or mock-infected (n=3), and monitored up to 7 dpi. Weight, blood glycemia, and clinical signs were determined. (**B**) Weight difference between B6 DIO and B6 lean mice before infection. (**C**) Glucose concentration measured in the blood of fasted B6 DIO and B6 lean mice before infection. (**D**) Body weight change of B6 DIO and B6 lean mice after infection. (**E**) Glucose concentration measured in the blood of mock-infected and SARS-CoV-2-infected B6 DIO and B6 lean mice at 2 dpi, 4 dpi, and 7 dpi. Bars represent the mean ± standard deviation. *, *P* ≤ 0.05; **, *P* ≤ 0.01; ***, *P* ≤ 0.001.

**
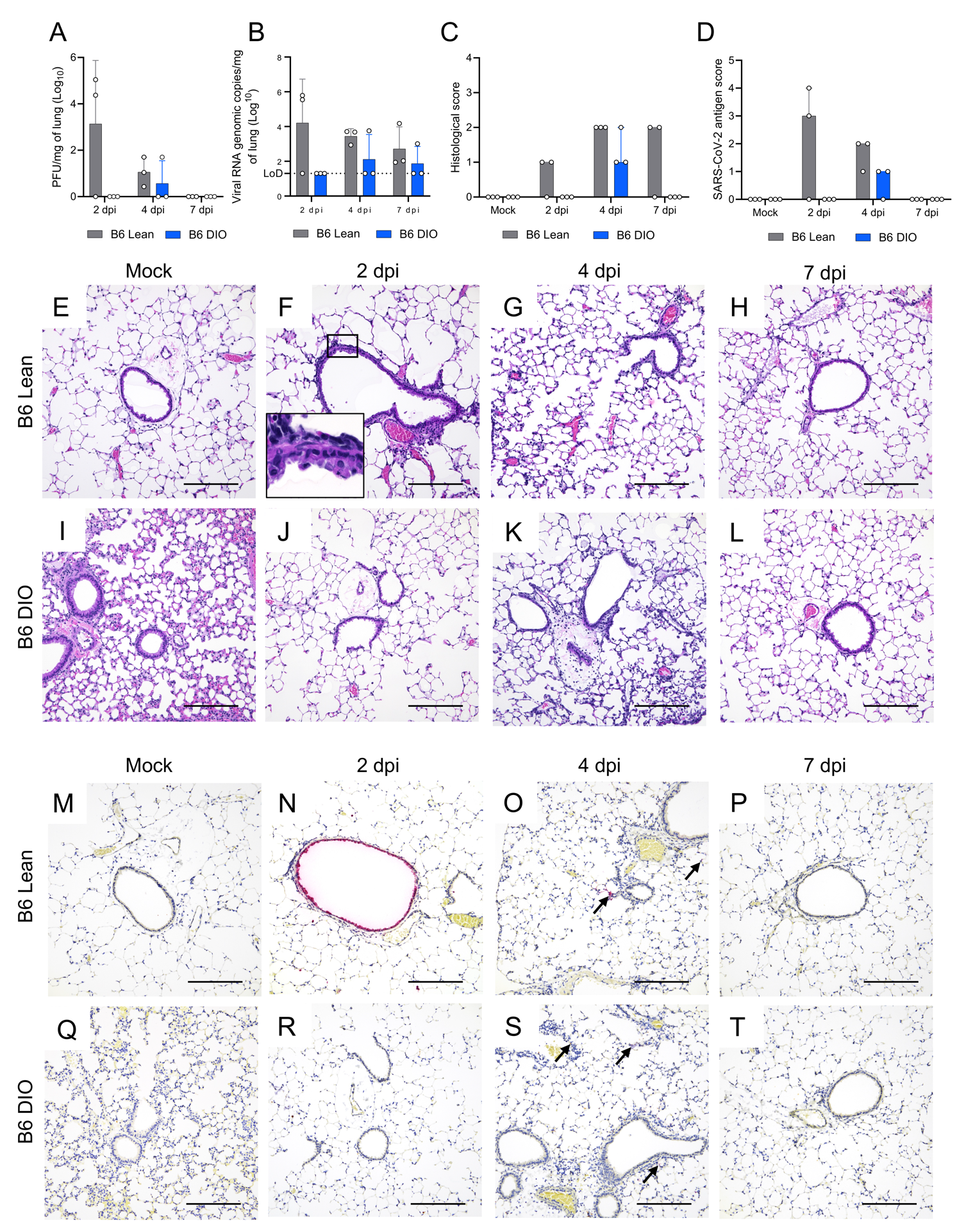
**

**Supplementary Figure 2.** Comparative temporal analysis of SARS-CoV-2 MA10 replication and pathological alterations in the lung of B6 DIO and lean mice. (**A**) Infectious viral particles and (**B**) viral RNA were quantified in the lungs of infected mice at 2, 4, and 7 dpi (n=3 per time point). The dotted line represents the limit of detection (LoD). Bars represent the mean ± standard deviation. (**C**) Histological scores and (**D**) SARS-CoV-2 antigen score in the lung of B6 DIO and B6 lean mice (n=3 per time point). Bars represent the median ± range. Temporal histologic lesions (H&E) (**E** – **L**) and viral antigen (Fast Red) abundance and distribution (**M** – **T**) in the lung of B6 DIO and B6 lean mice. At 2 dpi, bronchiolar epithelial necrosis was evident in B6 lean mice (**F**, inset). At 4 dpi, there is mild interstitial pneumonia in both B6 DIO and lean mice (**H** and **I**). Viral antigen was identified in bronchiolar epithelial cells at 2dpi in B6 lean mice (**N**), with sporadic antigen within rare alveolar epithelial cells at 4 dpi in both B6 DIO and lean mice (**O** and **S**, arrows). × 200 total magnification (Scale bar: 200 μm). *, *P* ≤ 0.05; **, *P* ≤ 0.01; ***, *P* ≤ 0.001.

**
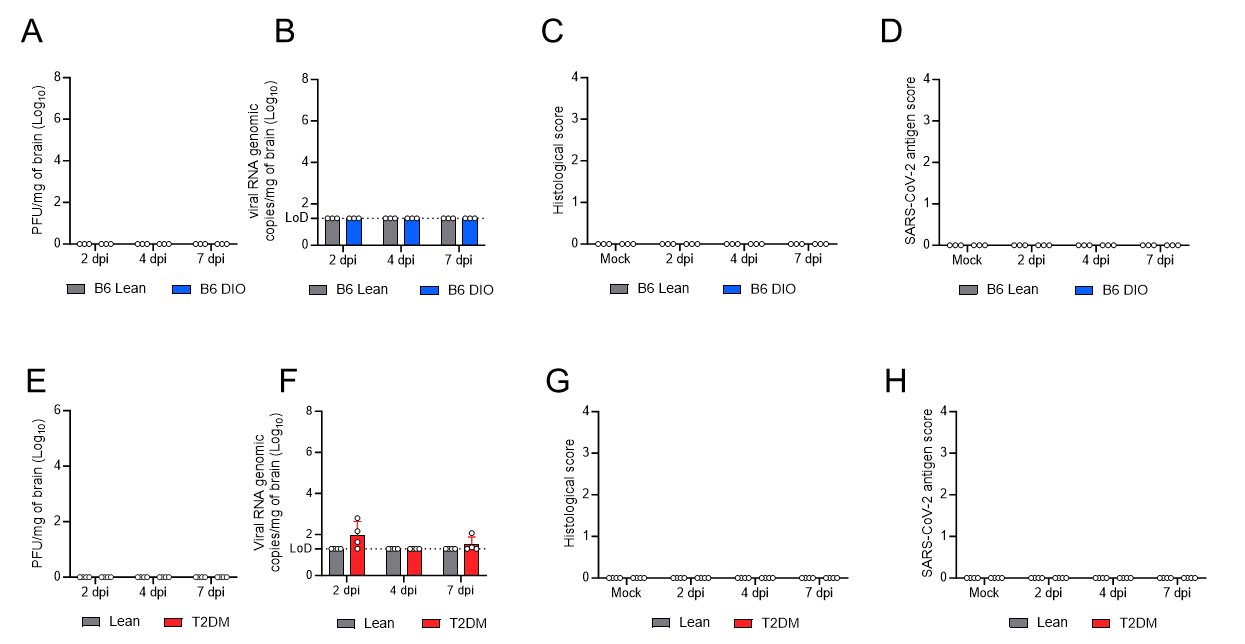
**

**Supplementary Figure 3. The SARS-CoV-2 MA10 strain does not exhibit neuro-invasion in B6 lean, B6 DIO, lean and T2DM, *Lepr*-deficient mice.** (**A**) Infectious viral particles, (**B**) viral RNA, (**C**) histological score and (**D**) SARS-CoV-2 antigen score measured in the brain of mock-infected and SARS-CoV-2 infected B6 DIO and B6 lean mice at 2 dpi, 4 dpi and 7 dpi. (**E**) Infectious viral particles, (**F**) viral RNA, (**G**) histological score, and (**H**) SARS-CoV-2 antigen score measured in the brain of mock-infected and SARS-CoV-2 infected lean and *Lepr*-deficient, T2DM mice at 2 dpi, 4 dpi and 7 dpi. Bars represent the mean ± standard deviation. *, *P* ≤ 0.05; ** *P* ≤ 0.01; *** *P* ≤ 0.001.

**
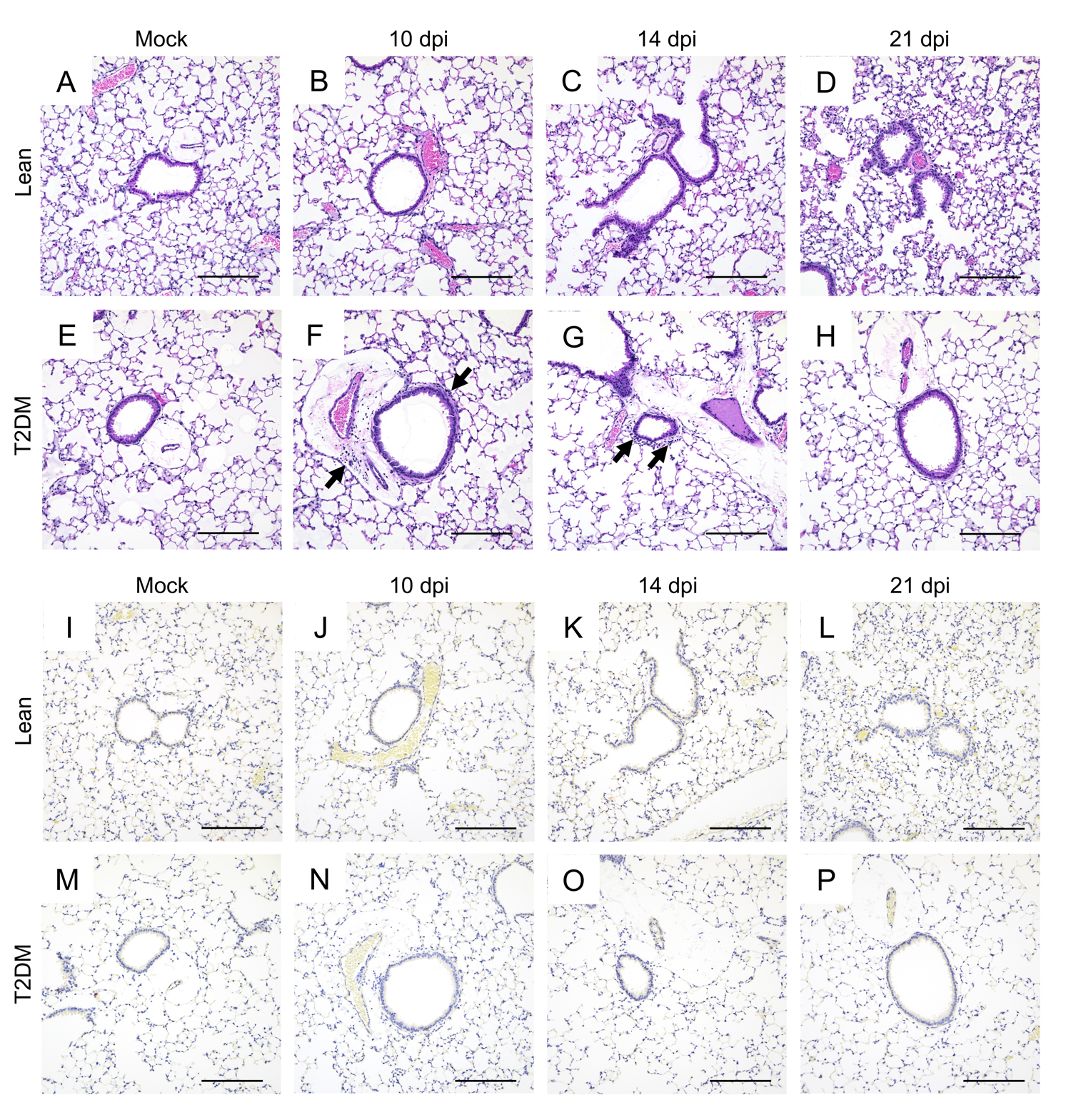
Supplementary Figure 4. Comparative temporal analysis of SARS-CoV-2 MA10 strain pathological alterations and tropism in the lung of lean and T2DM, *Lepr*-deficient mice at 10 dpi, 14 dpi, and 21 dpi.** (**A** - **H**) Temporal histological lesions (H&E) and (**I** - **P**) viral antigen (Fast Red) abundance and distribution in the lung of lean and T2DM, *Lepr*-deficient mice at 10 dpi, 14 dpi, and 21 dpi. Small numbers of mononuclear cells (lymphocytes) infiltrate perivascular and peribronchiolar regions in *Lepr*-deficient, T2DM mice at 10 dpi and 14 dpi (**F** and **G**, arrows). No viral antigen was detected (**I** - **P**). × 200 total magnification (Scale bar: 200 μm).

**
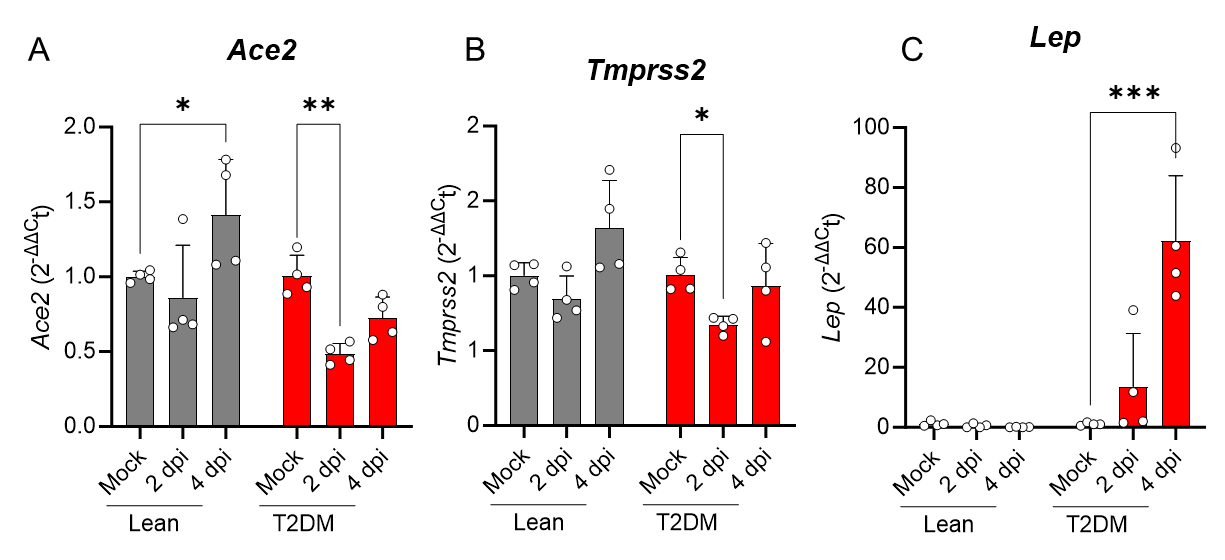
**

**Supplementary Figure 5. Relative gene expression of (A) *Ace2* (B) *Tmprss2* and Leptin (*Lep*) (C) in the lung of mock- and SARS-CoV-2 infected *Lepr*-deficient, T2DM mice and their lean counterparts.** Transient downregulation of *Ace2* and *Tmprss2* expression was observed in the lung of T2DM, *Lepr*-deficient mice at 2 dpi, while an exacerbated increase of *Leptin (Lep)* expression was observed in the lung of *Lepr*-deficient, T2DM mice at 4 dpi. Bars represent the mean ± standard deviation. *, *P* ≤ 0.05; ** *P* ≤ 0.01; *** *P* ≤ 0.001.

**
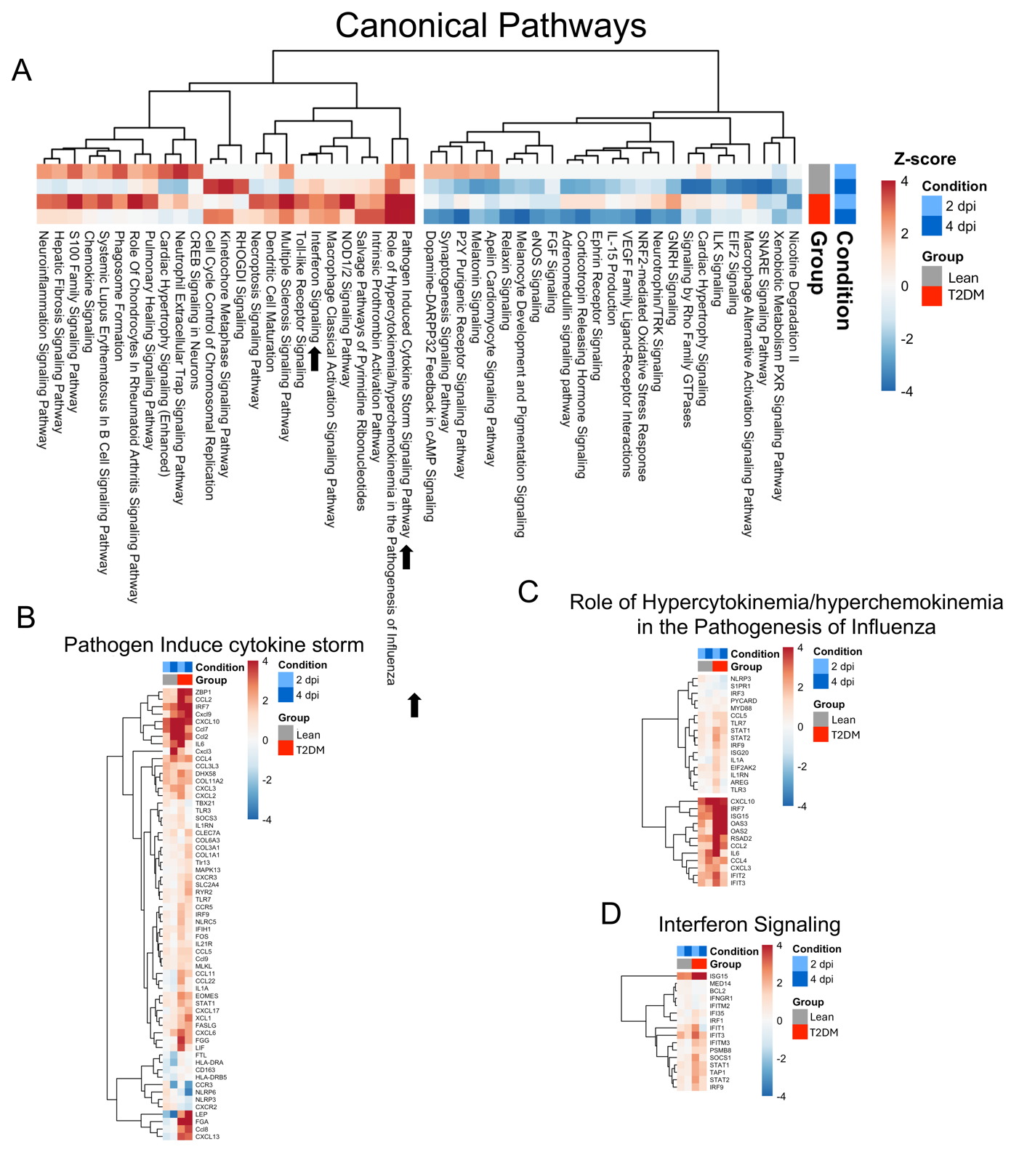
Supplementary Figure 6. Ingenuity Pathway Analysis (IPA) of differentially expressed genes.** Fold change values of DEGs of Mock- and SARS-CoV-2-infected *Lepr*-deficient, T2DM mice and their lean counterparts at 2 dpi and 4 dpi were analyzed on the IPA software and sorted into canonical pathways. (**A**) heatmap showing the top 25 up-regulated and down-regulated canonical pathways identified by Z-score. Z-scores of ≥ 2 or ≤ -2 are considered significant. The back arrows indicate the canonical pathways presented in **B** - **D**. Heatmaps showing the fold change of the genes belonging to the IPA pathways (**B**) “Pathogen Induce cytokine storm”, (**C**) “Role of hypercytokinemia/hyperchemikinemia in the Pathogenesis of Influenza” and (**D**) “Interferon Signaling”.

**
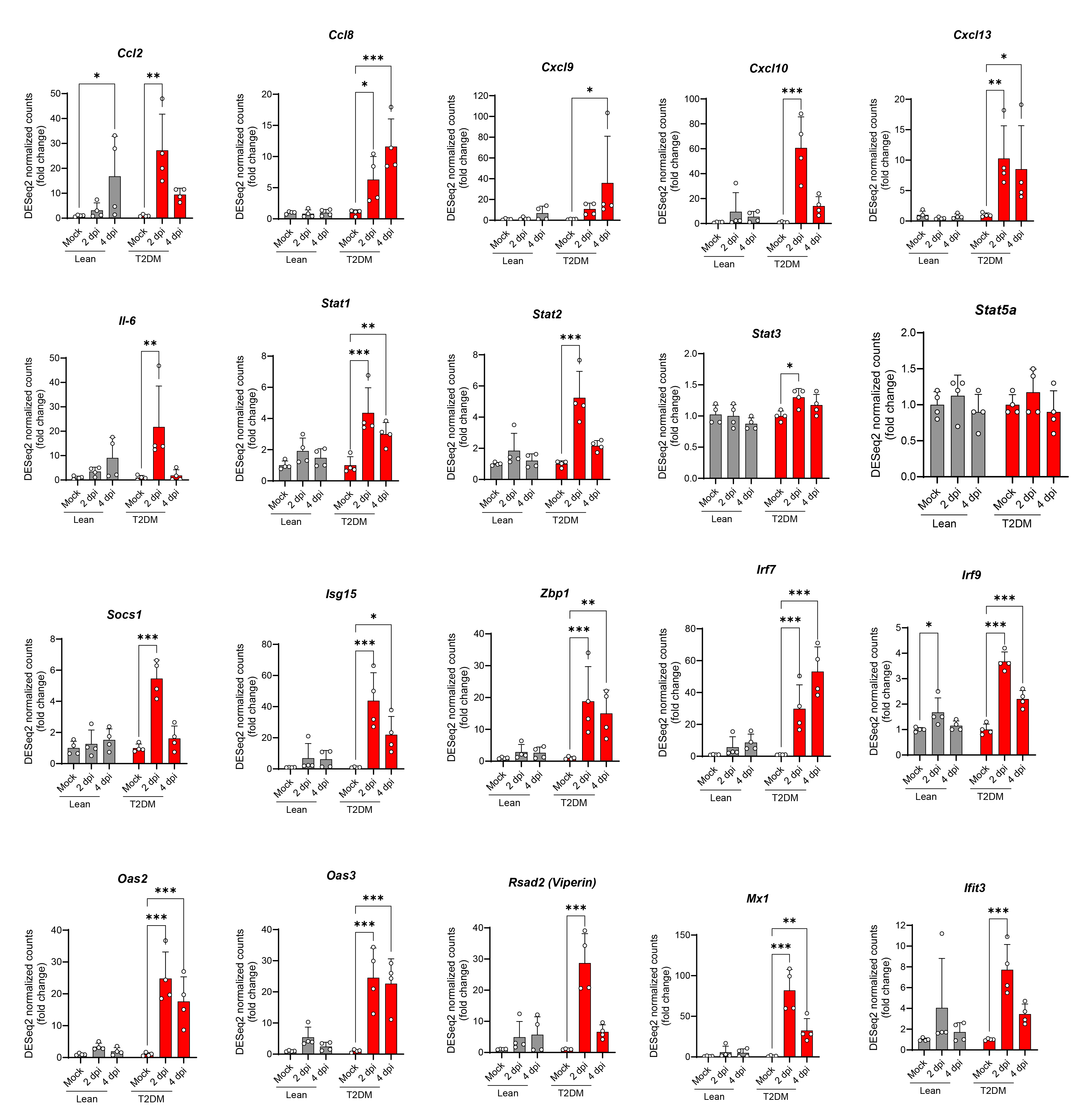
**

**Supplementary Figure 7. Fold change of the top genes related to the cytokine storm and type I interferon response pathways measured by DESeq2.** Bars represent the mean ± standard deviation. *, *P* ≤ 0.05; ** *P* ≤ 0.01; *** *P* ≤ 0.001.

**
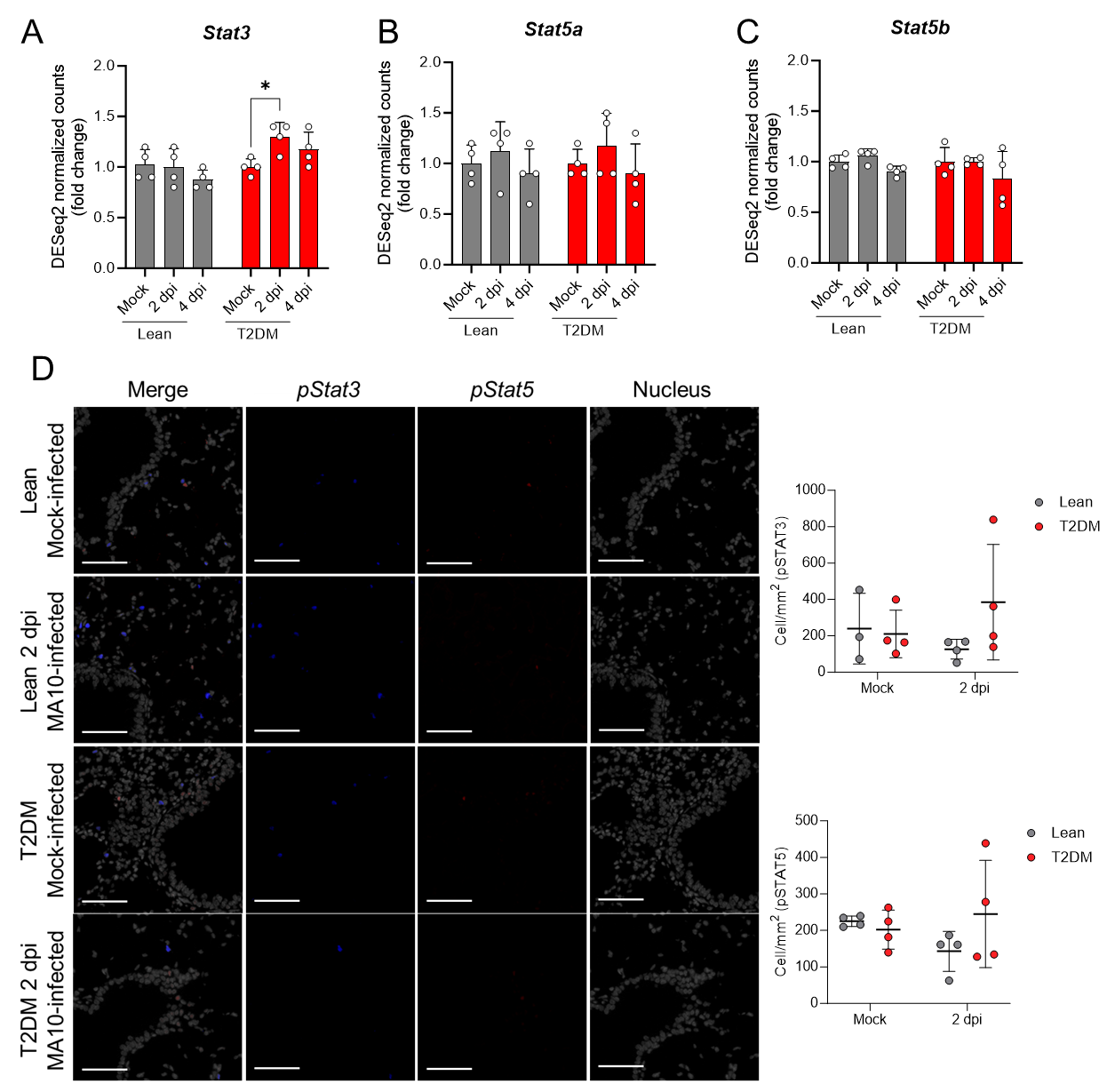
**

**Supplementary Figure 8.** *Lepr*-deficient, T2DM mice show no STAT3 or STAT5 activation following SARS-CoV-2 infection. Relative gene expression of *pStat3* (**A**), *pStat5a* (**B**), and *PStat5b* (**C**) was not up-regulated in the lung of mock-infected and SARS-CoV-2-infected lean and *Lepr*-deficient, T2DM mice at 2 dpi and 4 dpi. Multiplex immunofluorescence staining and quantification of pSTAT3 and pSTAT5 confirm the absence of their expression in the lungs of both mock-infected and SARS-CoV-2-infected lean and *Lepr*-deficient, T2DM mice at 2 dpi. (Scale bar: 100 μm). Bars represent the mean ± standard deviation. *, *P* ≤ 0.05; **, *P* ≤ 0.01; ***, *P* ≤ 0.001.

**
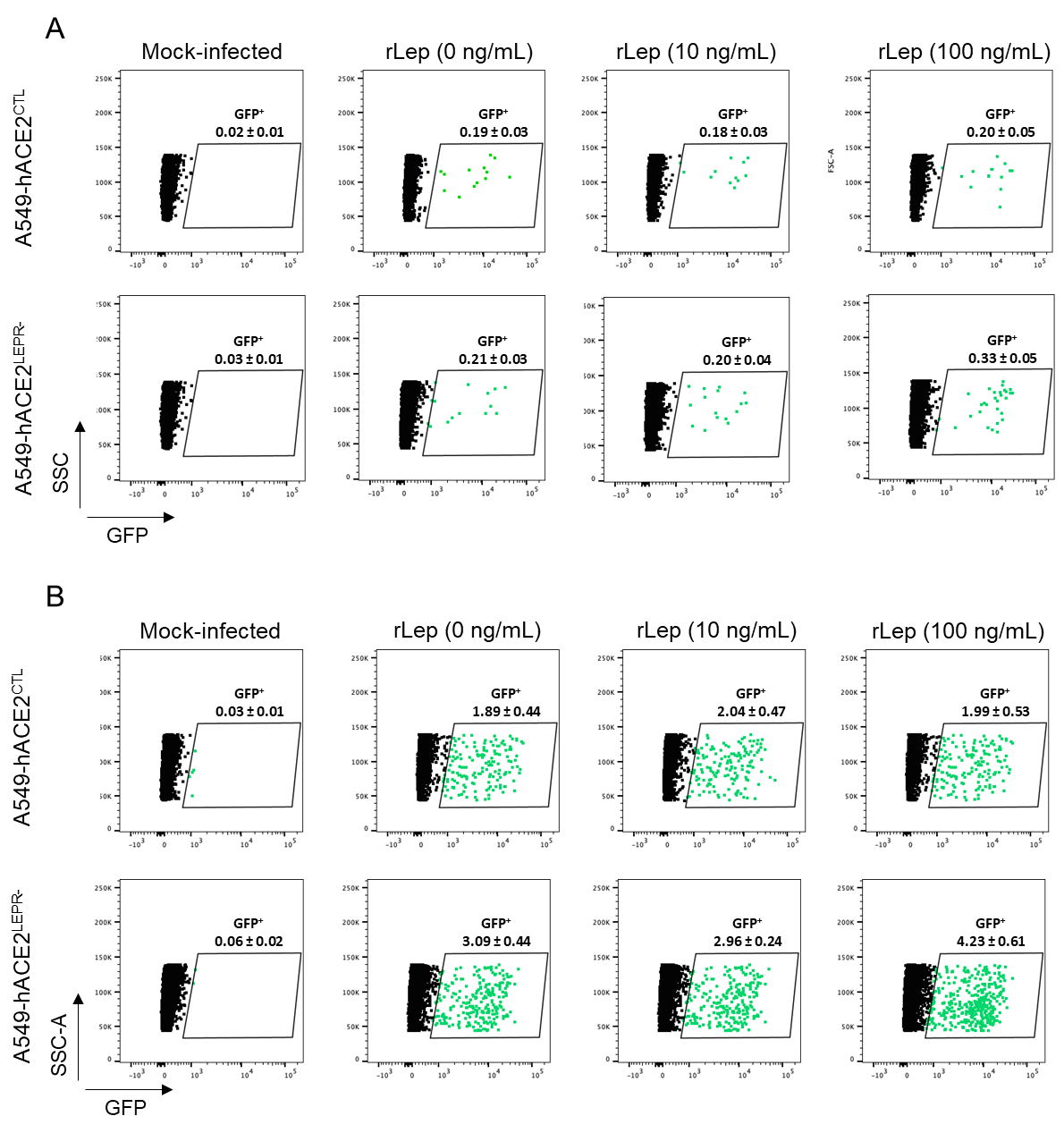
**

**Supplementary Figure 9. Representative FACS plots depicting gated leptin-treated A549-hACE2^CTL^ and A549-hACE2^LEPR-^ cells, either infected or uninfected with ic-SARS-CoV-2-eGFP, at 12 hpi (A) and 24 hpi (B).** The gates containing the green events correspond to cells infected with ic-SARS-CoV-2-eGFP. The number above the gate represents the percentage of total cells ± standard deviation within the gate. GFP, green fluorescent protein; SSC, side scatter.

## Supplementary Tables

**Supplementary Table 1:** Specific antibodies used for immunofluorescence and multiplex immunohistochemistry.

| **Target** | **Clone** | **Catalog number** | **Species** | **Reactivity** | **Dilution** | **Source** |
| --- | --- | --- | --- | --- | --- | --- |
| SARS-CoV-2 (Nucleoprotein) | 3A | N/A | Rabbit | SARS-CoV-2 | 1:5000 | Dr. J. A. Richt |
| SARS-CoV-2 (Nucleoprotein) | E8R5W | 68344 | Mouse | SARS-CoV-2 | 1:1000 | Cell Signaling |
| Phospho-Stat1 | 58D6 | 9167T | Rabbit | Mouse | 1:200 | Cell Signaling |
| Cxcl10 | N/A | BS-1502R | Rabbit | Mouse | 1:50 | Bioss Antibodies |
| Phospho-Stat3 | D3A7 | 9145T | Rabbit | Mouse | 1:200 | Cell Signaling |
| Phospho-Stat5 | C71E5 | 9314T | Rabbit | Mouse | 1:200 | Cell Signaling |
| LEPR | ARC0454 | MA5-35247 | Rabbit | Human | 1:200 | Invitrogen |
| hACE2 | CL4035 | AMAB91262 | Mouse | Human | 1:500 | Sigma-Aldrich |

**Supplementary Table 2:** Primer sequences used for relative gene expression analysis.

| **Target (Accession number)** | **Forward primer (5’-3’)** | **Reverse primer (5’-3’)** | **Product length (bp)** |
| --- | --- | --- | --- |
| *Ace2* (NM_001130513.1) | GTCATGGATGCGCTTTGGAT | CTTGGGTTGGGCACTGCTTA | 88 |
| *Tmprss2* (NM_015775.2) | TGACGGGGTAGCACATTGTC | CACACGGGATACCAGGCTTT | 117 |
| *Lep* (NM_008493.3) | ACATACCGCATTTCAGGGCA | CCCAGGTATCCCGTGTCAAC | 70 |
| *Ifna1* (NM_010502.2) | ATTCTGCAATGACCTCCACCAG | CATCTTCCTGGGTCAGGGGAAAT | 95 |
| *Ifnb1* (NM_010510.2) | TGGGAGATGTCCTCAACTGC | CCAGGCGTAGCTGTTGTACT | 93 |
| *Ifng* (NM_008337.1) | AGCACTCGAATGTGTCAGGT | GCACCAGGTGTCAAGTCTCT | 76 |
| *Il-2* (NM_008366.3) | GAAACTCCCCAGGATGCTCA | CGCAGAGGTCCAAGTTCATCT | 99 |
| *Il-6* (NM_031168.2) | GGGACTGATGCTGGTGACAA | ACAGGTCTGTTGGGAGTGGT | 90 |
| *Ccl2* (NM_011333.3) | TGACCCCAAGAAGGAATGGG | ACCTTAGGGCAGATGCAGTT | 104 |
| *Cxcl10* (NM_021274.2) | CTGAGTCCTCGCTCAAGTGG | GTCGCACCTCCACATAGCTT | 69 |
| *Actb* (NM_007393.5) | CACTGTCGAGTCGCGTCC | TCATCCATGGCGAACTGGTG | 89 |
| *Gusb* (NM_001357025.1) | GGCGATGGACCCAAGATACC | ACCCTTGGGATACCACAACT | 84 |
| *Rpl13a* (NM_001357025.1) | CCCACAAGACCAAGAGAGGC | CACCATCCGCTTTTTCTTGTCA | 92 |
| GAPDH (NM_002046.7) | TCCAAAATCAAGTGGGGCGA | AAATGAGCCCCAGCCTTCTC | 89 |
| GUSB (NM_000181.4) | GATGCTGTACCCCCAGGAGA | GCGTCGGTTGTCAGAGAAGT | 91 |
| RPL13A (NM_012423.4) | AGGTATGCTGCCCCACAAAA | TGCCGTCAAACACCTTGAGA | 68 |
| LEPR (NM_002303.6) | TCTTGGTCCAGCCCACCATT | GCAGGGATGTAGCTGAGACAA | 115 |
| INFb1 (NM_002176.4) | ACGCCGCATTGACCATCTAT | GTCTCATTCCAGCCAGTGCT | 85 |

*Ace2*: Angiotensin converting enzyme 2; *Tmprss2*: transmembrane serine protease 2; *Lep*: Leptin; *Ifn*: Interferon; *Il*: Interleukin; *Ccl2*: C-C motif chemokine ligand *2; Cxcl10*: C-X-C motif chemokine ligand 10; *Actb*: Actin, beta; *Gusb*: Glucuronidase beta; *Rpl13a*: Ribosomal protein L13a; GAPDH: Glyceraldehyde-3-phosphate dehydrogenase; LEPR: Leptin receptor.
